# Chromatin regulator SMARCAL1 modulates cellular lipid metabolism

**DOI:** 10.1038/s42003-023-05665-6

**Published:** 2023-12-21

**Authors:** Taylor Hanta Nagai, Chrissy Hartigan, Taiji Mizoguchi, Haojie Yu, Amy Deik, Kevin Bullock, Yanyan Wang, Debra Cromley, Monica Schenone, Chad A. Cowan, Daniel J. Rader, Clary B. Clish, Steven A. Carr, Yu-Xin Xu

**Affiliations:** 1 Center for Genomic Medicine, Massachusetts General Hospital, Boston, MA 02114 USA; 2 Broad Institute of MIT and Harvard, Cambridge, MA 02142 USA; 3 Division of Cardiovascular Medicine, Beth Israel Deaconess Medical Center, Harvard Medical School, Boston, MA 02215 USA; 4 Division of Translational Medicine and Human Genetics, Perelman School of Medicine, University of Pennsylvania, Philadelphia, PA 19104 USA; 5 Institute for Translational Medicine and Therapeutics, Perelman School of Medicine, University of Pennsylvania, Philadelphia, PA 19104 USA; 6 Department of Medicine, Harvard Medical School, Boston, MA 02115 USA

**Keywords:** Mechanisms of disease, Molecular medicine

## Abstract

Biallelic mutations of the chromatin regulator SMARCAL1 cause Schimke Immunoosseous Dysplasia (SIOD), characterized by severe growth defects and premature mortality. Atherosclerosis and hyperlipidemia are common among SIOD patients, yet their onset and progression are poorly understood. Using an integrative approach involving proteomics, mouse models, and population genetics, we investigated SMARCAL1’s role. We found that SmarcAL1 interacts with angiopoietin-like 3 (Angptl3), a key regulator of lipoprotein metabolism. In vitro and in vivo analyses demonstrate SmarcAL1’s vital role in maintaining cellular lipid homeostasis. The observed translocation of SmarcAL1 to cytoplasmic peroxisomes suggests a potential regulatory role in lipid metabolism through gene expression. SmarcAL1 gene inactivation reduces the expression of key genes in cellular lipid catabolism. Population genetics investigations highlight significant associations between SMARCAL1 genetic variations and body mass index, along with lipid-related traits. This study underscores SMARCAL1’s pivotal role in cellular lipid metabolism, likely contributing to the observed lipid phenotypes in SIOD patients.

## Abstract (web summary)

This study presents in vitro, in vivo, and human genetic evidence showing that the chromatin regulator SmarcAL1 plays an indispensable role in regulating cellular lipid metabolism, a mechanism linked to the lipid phenotypes seen in SIOD patients.

## Introduction

SMARCAL1 (also known as SWI/SNF-related matrix-associated actin-dependent regulator of chromatin subfamily A-like protein 1) belongs to the SNF2 (sucrose non-fermenting type 2) motor protein family^1,2^. The protein consists of multiple domains that share homology with HepA-related protein (HARP), an ATP-dependent annealing helicase, and DNA-coupled ATPase. SMARCAL1 plays important roles in diverse cellular processes, including chromatin assembly and remodeling, transcription regulation, and DNA damage repair^3^.

Biallelic loss-of-function mutations in the SMARCAL1 gene are responsible for Schimke Immunoosseous Dysplasia (SIOD, MIM 242900), an autosomal recessive disorder characterized by severe growth defects and premature death^4,5^. SIOD is a multisystemic disease affecting various organs. Its primary clinical features include defective chondrogenesis, skeletal dysplasia, renal failure, and immunodeficiency. Notably, about half of SIOD patients experience cardiovascular complications, including transient ischemic attacks, stroke, and myocardial infarction, which are presumed to be associated with cerebral and coronary ischemia^5–7^.

Premature atherosclerosis is a notable phenotype frequently seen in SIOD patients, yet it remains relatively understudied^6^. This condition is prevalent and has been identified to impact the arteries of vital organs, including the brain, heart, and lungs. In these patients, arterial plaques exhibit classic characteristics of atherosclerosis, including focal lipid deposition, intimal and medial hyperplasia, macrophage infiltration, and the formation of foam cells.

Furthermore, hyperlipidemia is frequently observed in individuals with SIOD, in certain reported cases, exceptionally elevated levels of blood total cholesterol (TC) and triglyceride (TG) have been documented (e.g., TC, up to 915 mg/dl; TG, 215 mg/dl; and low-density lipoprotein cholesterol (LDL-C), 220 mg/dl)^8,9^. In specific tissues, there have been reports of fat accumulation, such as extensive adipose deposition found between myocytes within the hearts of SIOD patients^5^. The leading cause of premature atherosclerosis is likely hyperlipidemia, although the mechanisms underlying its development remain poorly understood.

SMARCAL1, a chromatin regulator, forms complexes with replicating protein A (RPA)^10–12^. The majority of research on SMARCAL1 has centered on its nuclear activities during DNA replication and replication-associated DNA damage repair^13^. However, recent studies have shed light on SMARCAL1’s additional role in regulating the transcription of numerous genes^14–16^. SMARCAL1 deficiency has been shown to affect gene expression in fibroblasts obtained from SIOD patients, resulting in abnormal responses to heat stress in both Drosophila and mice^16^. While the precise mechanisms governing SMARCAL1’s transcriptional regulation remain unclear, further studies in this area could yield important insights into its biological functions.

Here, using a proteomic approach, we discovered that SmarcAL1 (referring to the gene name in mice and rats) interacts with angiopoietin-like 3 (Angptl3), a well-known lipoprotein regulator, suggesting a potential role for SmarcAL1 in lipid metabolism. Deleting the SmarcAL1 gene in cell models down-regulated the expression of many genes essential for cellular lipid catabolism, leading to a significant accumulation of TGs and fatty acids (FAs). In mice, the knockout (KO) of the SmarcAL1 gene resulted in elevated blood TG levels. Our findings are reinforced by population genetics studies, which show significant association between genetic variations at the SmarcAL1 gene locus and both body mass index (BMI) and various lipid-related traits. Our study unveils an unexpected facet of SMARCAL1’s role in lipid metabolism, which is vital for normal growth and development. Disruption of this function could potentially contribute to the development of SIOD.

## Results

### Proteomic analysis revealed that SmarcAL1 interacts with Angptl3

ANGPTL3 is a key lipoprotein regulator^17^. In our investigation of potential cellular targets for Angptl3, we utilized the McArdle-RH7777 (McA) rat liver cell line as a cell model. We generated McA cell lines stably expressing either an Fc tag or Fc-tagged human Angptl3 (Fc-ANG3) with its secretion signal at their N-termini. Notably, while the expression of endogenous Angptl3 (rat) was extremely low (barely detectable by Western analysis, Figs. S1, top and S8A), the stable Fc- and Fc-Angptl3-expressing cell lines provide a valuable model system for proteomic analyses aimed at identifying Angptl3-interacting proteins. Western analysis confirmed the efficient expression of Fc and Fc-Angptl3 (Figs. S1 and S8).

**Fig. 1:**
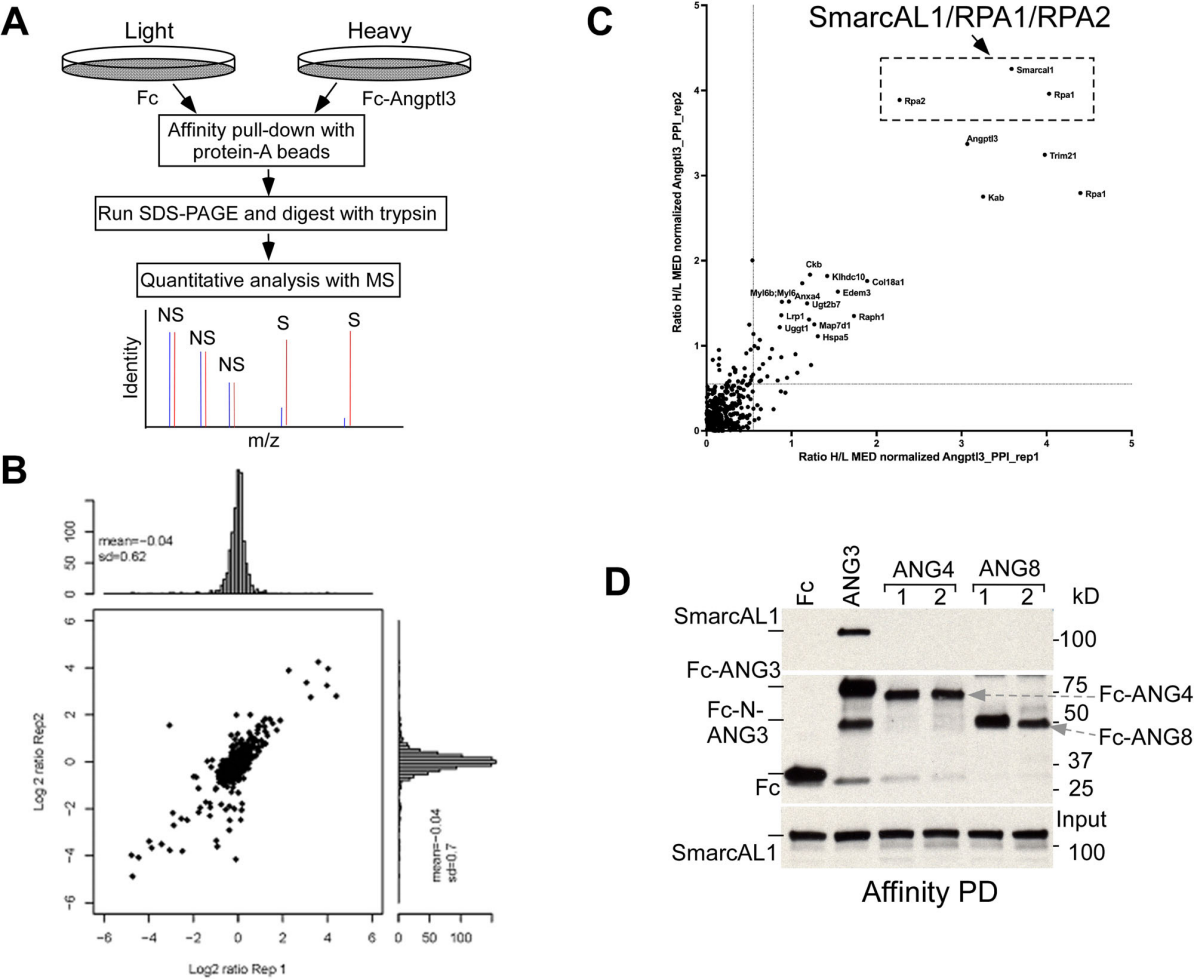
SmarcAL1 interacts with lipoprotein regulator Angptl3. **A** Schematic representation of SILAC (Stable Isotope Labeling by Amino acids in Cell culture) proteomics workflow. **B** Proteomic analysis of Angptl3-interacting proteins. Extracts of Fc and Fc-Angptl3 McA cells (two replicates for each cell line) (as in Fig. S1) labeled with modified amino acids were incubated with protein-A beads for affinity pull-down (PD) assays. Precipitates were subjected for SILAC proteomic analysis. X and Y axes represent log2 ratios for Fc-Angptl3- vs. Fc-interacting individual proteins for replicates 1 and 2, respectively. **C** Scatter plot represents log2 ratio change of proteins specifically co-PDed with Fc-Angptl3. Each dot represents a single protein. **D** SmarcAL1 specifically interacts with Angptl3, but not with Angptl4 and Angptl8. Western analysis of the protein-A affinity precipitates from Fc, Fc-Angptl3, -Angptl4, and -Angptl8 cells with anti-SmarcAL1 (top and bottom) and HRP-conjugated anti-Fc (middle) antibodies. See Fig. S9 for full images of this panel.

We used an unbiased quantitative proteomic approach known as Stable Isotope Labeling by Amino acids in Cell culture (SILAC) to identify proteins that interact with Angptl3^18,19^. In this method, Fc and Fc-Angptl3 cells were metabolically labeled with modified amino acids (Fig. 1A). Protein-A affinity pull-down (PD) was then used to precipitate the labeled Fc and Fc-Angptl3 and its interacting partners^20,21^. Figure 1B illustrates that the interacting proteins are well represented in the two independent replicates. The results show that Angptl3 selectively associates with specific cytoplasmic proteins (Fig. 1C). However, the most abundant interacting proteins identified were the nuclear SmarcAL1 complex containing RPA1 and RPA2^13^. To assess the specificity of this interaction, we generated two additional McA cell lines expressing either Fc-Angptl4 or Fc-Angptl8. Affinity PD analyses demonstrated that SmarcAL1 exclusively interacts with Angptl3, but not with Angptl4 and Angptl8 (Figs. 1D and S9)^22–24^. The specific binding of Angptl3 with the SmarcAL1/RPA complex suggests a functional significance in lipid metabolism.

### SmarcAL1 is indispensable for maintaining cellular lipid homeostasis

**Fig. 2:**
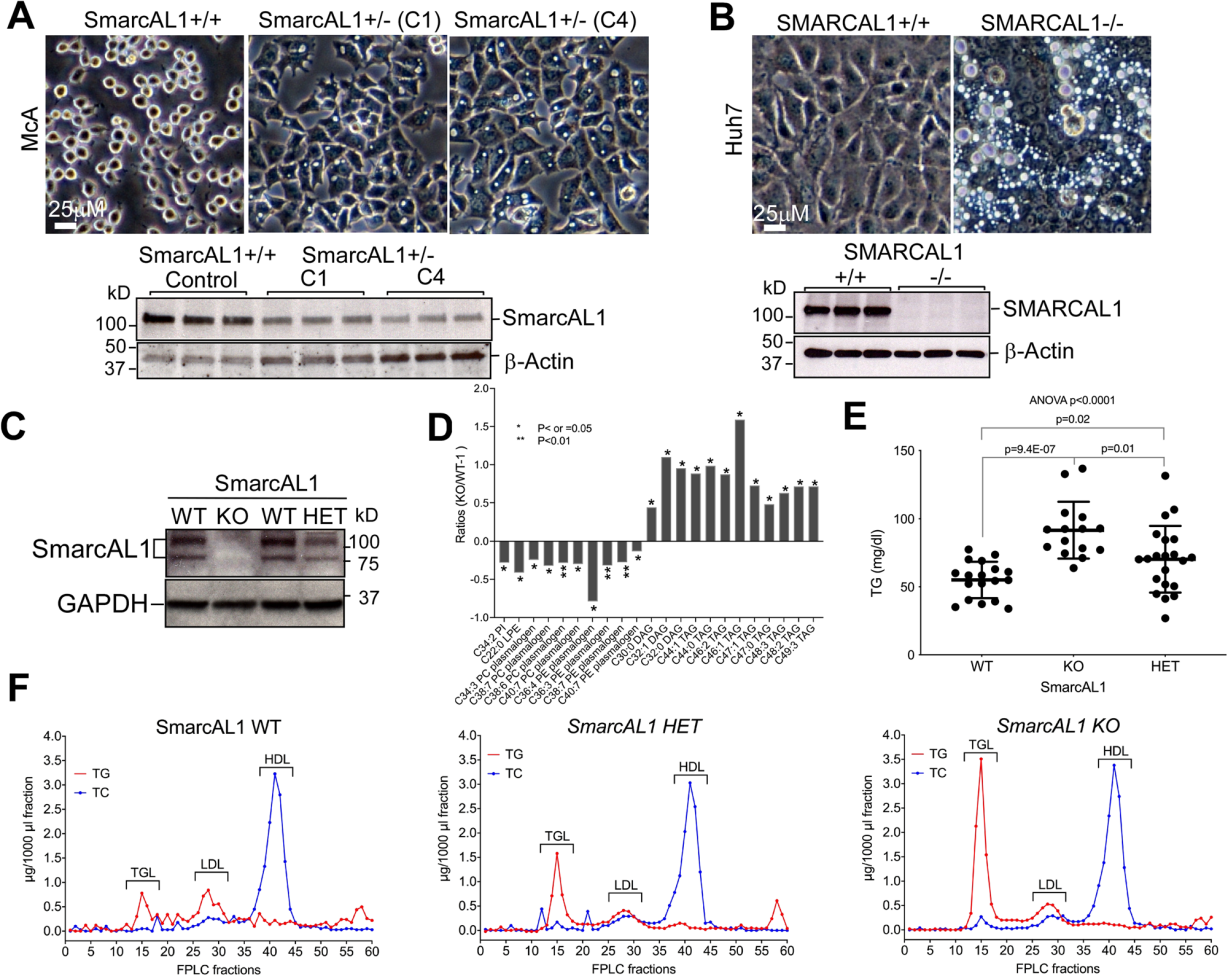
Inactivation of SmarcAL1 gene in cells disrupts cellular lipid homeostasis and SmarcAL1 KO in mice drastically increases plasma triglyceride (TG) levels. **A**, **B** Inactivation of SmarcAL1 gene in cells induces massive lipid droplet (LD) formation. Heterologous (**A**) and homologous (**B**) inactivation of SmarcAL1 genes in McA (**A**) and Huh7 (**B**) cells, respectively, induced LD formation captured under light microscopy (top). SmarcAL1 expression was confirmed with Western analysis (three replicates for each cell line) with anti-SmarcAL1 and -β-actin antibodies (bottom). C1 and C4 in A represent two McA clones with heterologous inactivation of SmarcAL1 gene. **C** Confirmation of SmarcAL1 heterozygous (HET) or homozygous (KO) gene deletion in mice. Western analysis of SmarcAL1 expression of liver extracts from the mice as indicated using anti-SmarcAL1 and -GAPDH antibodies. See Figs. S10–S12 for full images of the Western blots. **D** Metabolomic analysis of plasma from the WT and KO littermates (3 mice each). Ratios were calculated by comparing the averages of each metabolite of KO mice with those of WT mice and minus one. **E**, **F** Inactivation of SmarcAL1 gene increases plasma TG levels in mice. Plasma TG levels from individual littermates of the WT, HET, and KO mice with normal chow diet. Individual plasma samples were measured for TG using TG colorimetric assay kit (Cayman). Each dot represents a single mouse (**E**). Comparison of the plasma TG and total cholesterol (TC) profiles from the littermates of the WT, HET, and KO mice. Pooled plasma samples (from six mice each) from WT, HET and KO mice were fractionated by FPLC, and the fractions were analyzed for TG and TC separately. TG-rich lipoprotein (TRL), LDL and HDL in the fractions are indicated (**F**). *P* values calculated from *t* test and two-way ANOVA as indicated.

To investigate SmarcAL1’s role in lipid metabolism, we used a plasmid-based CRISPR/Cas9 system to inactivate the SmarcAL1 gene in McA cells (see Methods). After screening, we isolated two single cell clones (C1 and C4) exhibiting heterologous inactivation of the SmarcAL1 gene (SmarcAL1^+/−^) in the McA cells (Figs. 2A and S10). The SmarcAL1 mutations were confirmed using the T7 endonuclease I assay^25^. Western analysis indicate that SmarcAL1 protein level was greatly reduced (Figs. 2A, bottom, and S10). Interestingly, under standard growth conditions, the mutant cells displayed a notable accumulation of lipid droplets (LDs), a phenotype not observed in the control cells (Fig. 2A, top).

To reproduce this phenotype in a different cell type, we used a lentivirus-based CRISPR/Cas9 system to generate homozygous inactivation of the SMARCAL1 gene (SMARCAL1^−/−^) in human hepatoma Huh7 cells (see Methods). Western analysis confirmed the successful inactivation (Figs. 2B, bottom, and S11). We isolated individual SMARCAL1^−/−^ and control clones, wherein the mutant clones exhibited a pronounced accumulation of LDs. Notably, these phenotypes in SMARCAL1^−/−^ Huh7 cells were much more obvious than those from the SmarcAL1^+/−^ McA cells (Fig. 2B versus 2 A, tops).

To investigate changes in cellular lipid metabolites in SmarcAL1^+/−^ McA and SMARCAL1^−/−^ Huh7 cells as compared to their respective wild-type (WT) controls, we conducted comprehensive metabolomics analyses. In alignment with the observed lipid phenotypes, we detected increased accumulation of TGs, FAs, plasmalogens, and various other lipid metabolites (Figs. S2 and S3, see Supplementary Data 1 and 2 for respective details). It is worth noting that certain distinctions emerged between the two cell types. For instance, in SMARCAL1^−/−^ Huh7 cells, we observed elevated TG levels with longer carbon chains and reduced TG levels with shorter carbon chains (Fig. S3A), whereas in SmarcAL1^+/−^ McA cells, we observed increases in all TG metabolites (Fig. S2A). Across both cell types, an increased accumulation of FAs was consistently observed. Collectively, our data indicate that SmarcAL1 deficiency disrupts cellular lipid metabolism, underscoring the important role of SmarcAL1 in maintaining cellular lipid homeostasis.

To provide in vivo evidence, we generated SmarcAL1 gene deletion mice using the CRISPR/cas9 genome editing system (see Methods). After extensive screening, we obtained one strain (SmarcAL1^−/−^) with confirmed SmarcAL1 gene deletion and null protein expression (Figs. 2C, and S12). Our initial body weight analysis did not show significant changes among the littermates of the WT, heterozygous (HET) and homozygous (KO) gene deletion mice (Fig. S4A). Blood TG tests unveiled elevated plasma TG levels in both the HET and KO mice, with a more increase observed in the KO mice when compared to their respective WT littermates (Fig. 2E). Plasma FPLC analysis confirmed a significant rise in TG-rich lipoprotein (TRL) levels in both the KO and HET mice (Fig. 2F), while TC, high-density lipoprotein cholesterol (HDL-C), LDL-C, and glucose levels exhibited no apparent changes (Fig. S4B–F). It’s worth noting that there is a discrepancy in the TG levels between Fig. 2E, F. This variance could be attributed to variations in the measuring methods used, as well as limitations arising from the exclusion of the mice in the FPLC assay due to constraints related to plasma sample availability.

Furthermore, mass spectrometric analysis of plasma lipid metabolites showed an increase in neutral lipids, such as TG and diglycerides (DG), along with a decrease in the levels of membrane lipids, including phosphatidylethanolamine (PE) and phosphatidylcholine (PC) plasmalogens, in KO mice when compared to their WT counterparts (Fig. 2D). This suggests an enlargement in the sizes of TRL particles. It is noteworthy that the KO mice did not show the apparent change in blood cholesterol levels, which is an important lipid phenotype observed in SIOD patients^8,9^. We hypothesized that this discrepancy may be due to differences in lipid metabolism between humans and mice as previously confirmed^26,27^ (see Discussion).

The elevated plasma TG levels could be attributed to several factors. In vitro assays have revealed that the homozygous inactivation of the SMARCAL1 gene in Huh7 cells effectively blocked the uptake of Dil-very low-density lipoproteins (Dil-VLDLs) (Fig. S5A, B). This finding is supported by the reduced LRP1 expression in the McA cells upon SmarcAL1 gene inactivation (see below), aligning with the high blood TG levels observed in SmarcAL1 KO mice (Fig. 2E, F). While these cells also exhibited decreased secretion of VLDL particles (Fig. S5C, D), we reasoned that the slowed or reduced uptake of TRLs, specifically those derived from dietary fat, may exert a more pronounced impact on blood TG levels than the reduction in secretion. Nonetheless, further in vivo investigations are necessary to confirm this hypothesis. Taken together, the above results underscore the significant role of SmarcAL1 in regulating cellular lipid metabolism, particularly in modulating blood TG levels within the mouse model.

### SmarcAL1 translocates from nuclei to cytoplasmic peroxisomes

**Fig. 3:**
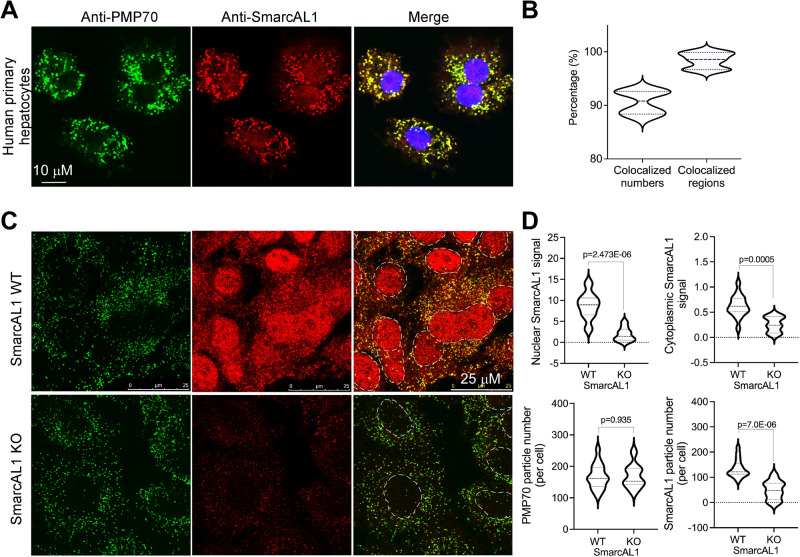
SMARCAL1 translocates from nucleus to cytoplasm and enriches on peroxisomes. **A** Enrichment of SMARCAL1 on peroxisomes. Human primary hepatocytes were fixed and hybridized with anti-PMP70 (peroxisome marker, green) and -SmarcAL1 (red), and then with fluorescence-labeled secondary antibodies. Images were acquired with confocal microscopy. **B** Image quantification. Quantification was carried out from two independent experiments as in **A**. The averages and deviations were calculated from ~200 human hepatocytes. **C** Immunofluorescence analysis of SMARCAL1 distribution in SMARCAL1 KO and WT Huh7 cells. The cells were analyzed as in **A**. **D** Image quantification. Quantification of PMP70 and SMARCAL1 signals in cytoplasm and nucleus from the images as in **C**. Quantification was carried out from three independent experiments. The averages and deviations were calculated from three clones of the KO and control cells (>3000 cells for each). *P* values were calculated with *t* test as indicated.

SmarcAL1 is a nuclear protein. Its interaction with cytoplasmic Angptl3 likely occurs in cytoplasm. To investigate this, we performed confocal immunofluorescence assays using a variety of cell types, including the cell lines that we generated. Our investigations confirmed the earlier observation that SmarcAL1 indeed presents in cytoplasm^15^. Interestingly, during our analysis, we noted the presence of numerous dot-like structures within the cytoplasm that exhibited a significant enrichment of SmarcAL1 signal. After conducting many rounds of experimentation, we identified that these dot-like structures bore a resemblance to peroxisomes within the cytoplasm. To validate this observation, we carried out a confocal immunofluorescence analysis with anti-PMP70 (a marker for peroxisomes)^28^ and anti-SmarcAL1 antibodies using human primary hepatocytes (Thermo Fisher). Our investigation verified the presence of SMARCAL1 in both the nucleus and the cytoplasm. Notably, we observed that the cytoplasmic SMARCAL1 exhibited co-localization with PMP70 within these dot-like structures (Fig. 3A, B).

To confirm that the signal observed at peroxisomes originated from SMARCAL1, we conducted a similar analysis using SMARCAL1 KO Huh7 cells as described in Fig. 2B (Fig. 3C, D). We hypothesized that the absence of SMARCAL1 would eliminate the signal at peroxisomes. In the WT control cells, we observed the presence of SMARCAL1 signal within the nuclei and cytoplasmic dot-like structures, where SMARCAL1 co-localized with PMP70 (Fig. 3C, top). In contrast, in the KO cells, we noted a significant reduction in the signal within both the nuclei and cytoplasmic peroxisomes (bottom). This observation supports the notion that the signal emanating from peroxisomes indeed originates from the SMARCAL1 protein. It is worth noting that the similar numbers of peroxisomes observed in both the WT and KO cells suggest that SMARCAL1 is not required for the assembly of peroxisomes (Fig. 3D). These findings offer evidence of SMARCAL1’s translocation to cytoplasm and its subsequent localization within peroxisomes.

### Inactivation of SmarcAL1 gene down-regulated the expression of many genes that are essential for cellular lipid metabolism

**Fig. 4:**
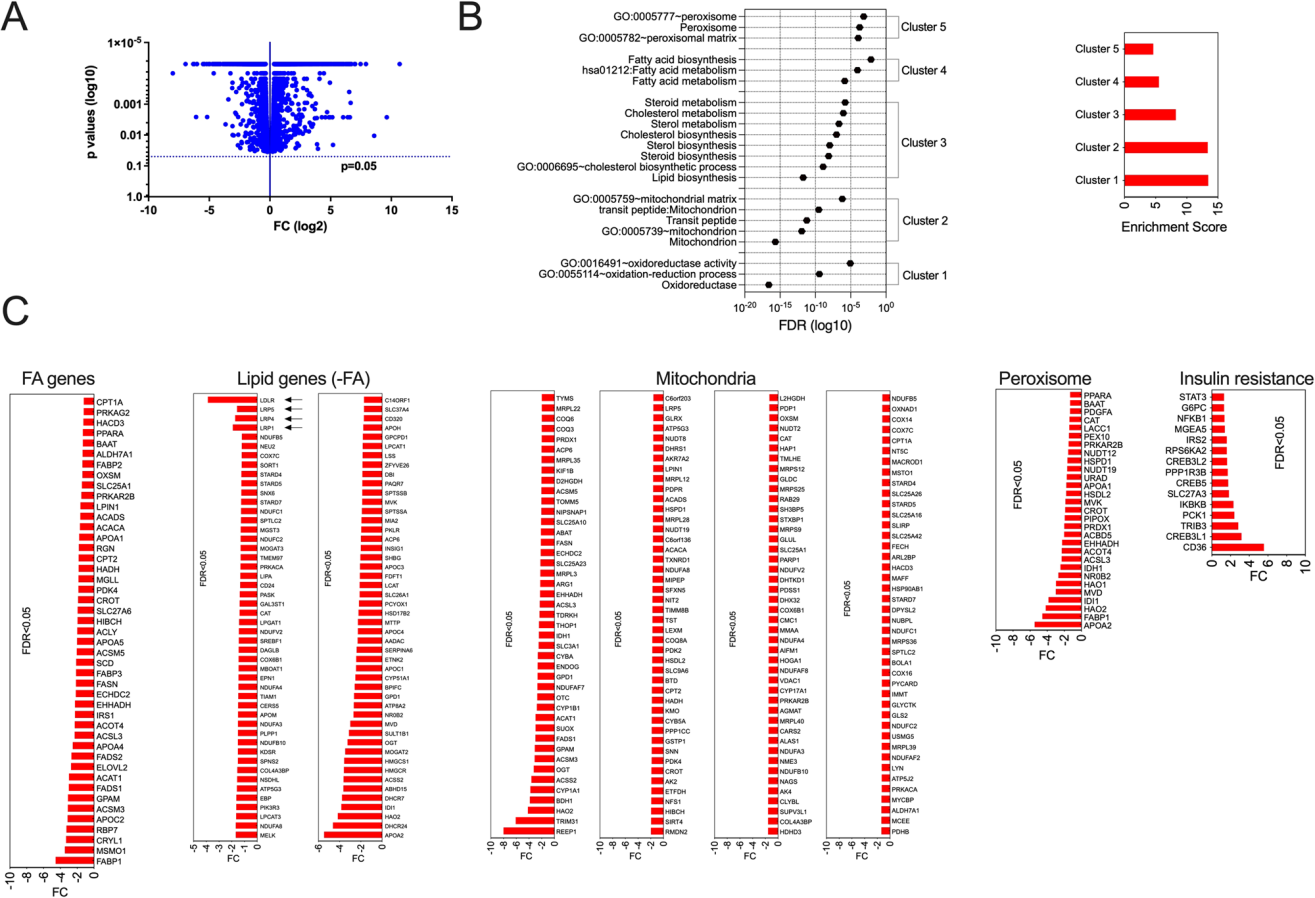
Inactivation of SmarcAL1 gene decreases the expression of genes related to cellular lipid metabolism. **A** Volcano plot for differential gene expression. Total RNA from SmarcAL1^+/−^ and WT control McA cells (three replicates for each) was used in RNA-seq assays. Differentially expressed genes (FDR < 0.05) were included in the plot. **B** Pathway and biological process enrichment analyses based on the differentially expressed genes. **C** Differentially expressed genes related to lipid, FA, mitochondria, peroxisome metabolism and insulin resistance. The expression of LDL receptor (LDLR), LDLR-related protein (LRP)1, LRP4 and LRP5 genes is indicated with arrows (see Supplementary Data 3 for expression details).

It is likely that SmarcAL1-regulated gene expression is the major mechanism underlying the lipid phenotypes observed. To investigate this, we conducted an RNA-seq assay to identify alterations in gene expression in cells with SmarcAL1 gene inactivation. We hypothesized that the heterologous deletion of SmarcAL1 in McA cells would provide a better cell model for understanding its role in transcription regulation, while its diminished expression would allow its functions in other cellular processes. Our results, presented in Fig. 4, demonstrate that the deletion significantly down-regulated multiple pathways and processes crucial for cellular lipid metabolism (Fig. 4B). Corresponding with these observations, the expression of many genes associated with mitochondria, peroxisomes, and FA catabolism (Fig. 4C and Supplementary Data 3) showed a notable decrease. Concurrently, there was an upregulation in the expression of genes associated with the insulin resistance pathway. These findings, coupled with the results from metabolomics analyses (Fig. S2), imply that the reduced gene expression and subsequent down-regulation of key lipid pathways and processes are the driving factors behind the observed lipid phenotypes.

It is important to note that the observed changes in gene expression are consistent with the lipid phenotypes seen in SIOD patients and the elevated blood TG levels observed in SmarcAL1 KO mice. Specifically, the inactivation of SmarcAL1 gene resulted in a significant reduction in the expression of several LDL receptor (LDLR) and LDLR-related protein (LRP) family genes, including LRP1, LRP4, and LRP5 (Fig. 4C).

The hepatic LRP1 expression is well-established as a critical factor in the clearance of TGLs^29,30^, including remnants of chylomicrons and VLDLs, which may elucidate the elevated TG levels seen in the KO mice. Furthermore, LDLR plays a pivotal role in determining blood cholesterol levels^31^. Although we did not observe changes in cholesterol levels in the SmarcAL1 KO mice (see Discussion), the downregulation of LDLR expression aligns with the hypercholesterolemia observed in SIOD patients.

### Tissue-specific expression of SMARCAL1 and phenotypes linked to genetic variations at the SMARCAL1 gene locus

**Fig. 5:**
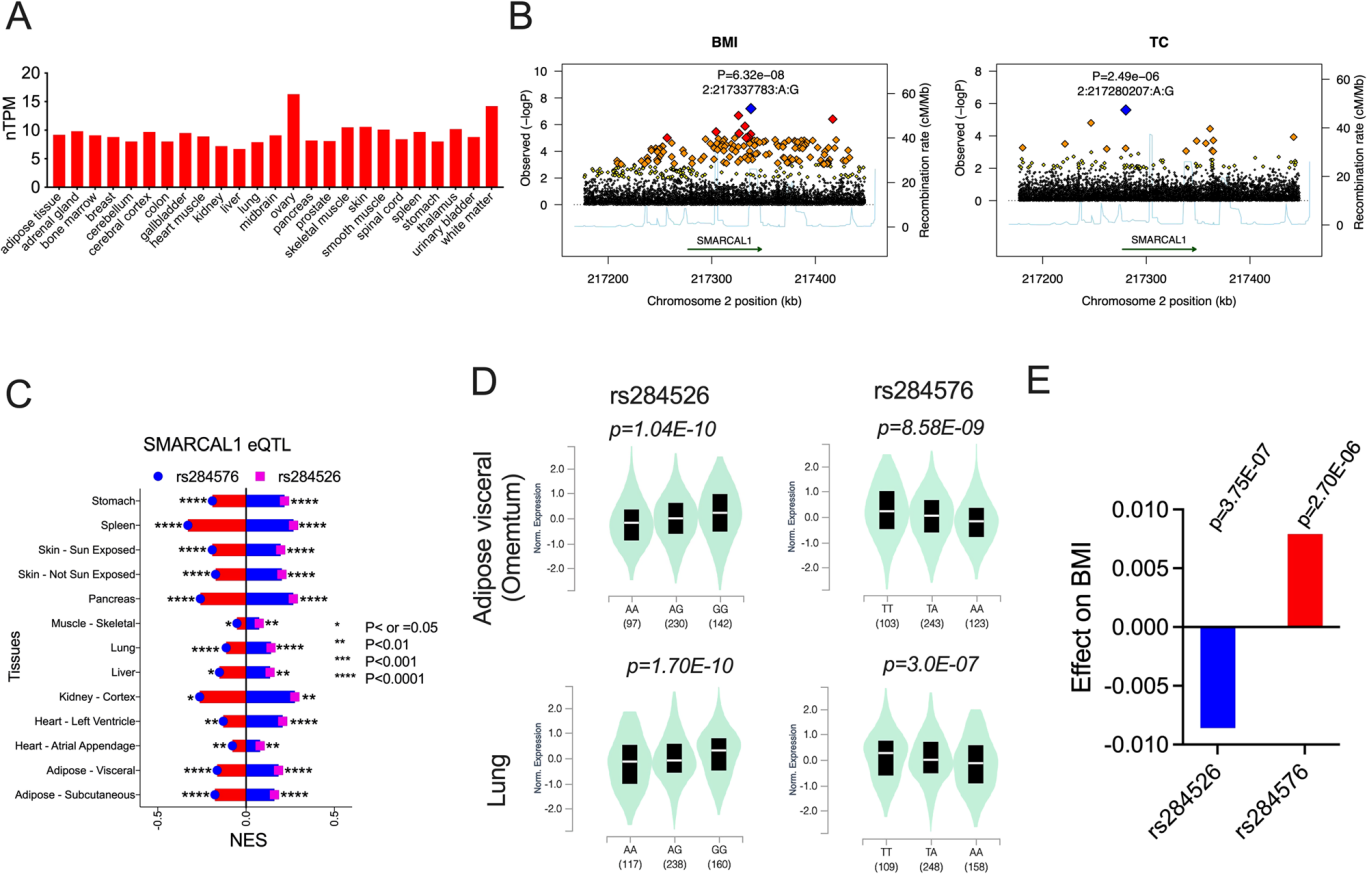
SMARCAL1 tissue-specific expression and association analysis of the genetic variations at SMARCAL1 gene locus. **A** SMARCAL1 tissue-specific RNA expression profile. The normalized RNA expression was adapted from a consensus dataset created from two independent sources, HPA (human protein atlas) and GTEx (genotype-tissue expression) RNA-seq data. nTPM, normalized transcript per million. **B** Genetic association analysis of the variants at the SMARCAL1 gene locus. Regional association plots of the variants with BMI (left) and total cholesterol (TC) (right). The *p* values and variant ID of the top variants (blue square) for each phenotype are indicated (see Figs. S6 and S7 and Supplementary Data 4–6 for the genetic association details). **C**–**E** SMARCAL1 eQTL (expression quantitative trait loci) analysis and its effect on BMI. Two variants, rs284526 and rs284576, have opposite effects on SMARCAL1 expression from GTEx database (included the major organs and tissues) (**C**). Violin plots represent the effect of rs284526 and rs284576 eQTLs on SMARCAL1 expression in the representative tissue and organ as indicated (**D**). The effect on BMI of the two variants based on association studies of GIANT-UK Biobank GWAS Meta-analysis (**E**).

SMARCAL1 exhibits ubiquitous expression across many tissues and organs. The tissue-specific RNA expression profile (Fig. 5A) was derived from a consensus dataset (courtesy of the Human Protein Atlas (HPA), www.proteinatlas.org). The results include normalized expression from 55 tissue types, incorporating data from both HPA^32^ and Genotype-Tissue Expression (GTEx)^33^ transcriptomics datasets. The expression data reveal that SMARCAL1 is highly expressed in several key tissues involved in energy storage and expenditure, including adipose tissues, muscles, and livers^3,16^.

To explore potential associations between genetic variations at the SMARCAL1 gene locus and lipid-related phenotypes, we collected data from population genetic studies based on bottom-line integrative analysis (https://a2f.org/)^34^. The approach allows to use pooling data from multiple studies to detect novel significant associations that might not be detectable at the level of individual datasets but become significant when multiple studies are included (see Methods). The results unveiled several variants linked to diverse human phenotypes, such as neutrophil count and fractional anisotropy in cerebral small vessel disease (FA in CSVD), with *p* values reaching genome-wide significance (<5.0 × 10^−8^) (Fig. S6A and Supplementary Data 4).

Importantly, these genetic variations also exhibited associations with metabolic syndromes, encompassing obesity, type 1 diabetes (T1D), type 2 diabetes (T2D), all diabetic kidney disease (DKD) vs. controls, hip circumference, and visceral abdominal tissue to abdominal subcutaneous adipose tissue (VAT to ASAT) ratio (Fig. S6B and Supplementary Data 5). Notably, the association of the leading variant with BMI approached genome-wide significance (Fig. 5B, left). Moreover, the gene locus is significantly linked to several common lipid phenotypes, including TC, TG, LDL-C, HDL-C, and non-HDL-C (Fig. 5B, right, Fig. S7 and Supplementary Data 6). The *p* values of the top variants associated with these lipid phenotypes either reached or fell just below the locus-wide significance threshold (<5.0 × 10^−4^).

To gain deeper insights into the impact of genetic variations on SMARCAL1 expression and its relationship with phenotypes, we curated data from both expression quantitative trait loci (eQTL) analysis and genetic studies. Our investigation revealed that two variants, rs284526 and rs284576, exert opposing effects on SMARCAL1 expression across various tissues. Specifically, rs284526 was associated with increased expression, whereas rs284576 was linked to decreased expression, as revealed by the GTEx database (Fig. 5C). Furthermore, single-tissue eQTL analyses demonstrated that rs284526 enhances tissue-specific SMARCAL1 expression, while rs284576 diminishes it (Fig. 5D)^33^.

Of notable interest, genetic association studies from GIANT-UK Biobank GWAS meta-analysis show that rs284526 is significantly linked to the reduced BMI, whereas rs284576 is associated with increased BMI^35^ (Fig. 5E). These findings imply that differential SMARCAL1 expressions could indeed influence human phenotypes. Although the *p* values for these associations only fall below the genome-wide significance threshold, the consistency of our results in this study, coupled with the lipid phenotypes observed in SIOD patients, suggests that the connections between SMARCAL1 variants and these phenotypes are likely causal.

Collectively, all the data presented in this study support the notion that SMARCAL1 plays an indispensable role in cellular lipid metabolism, and aberrant expression of its gene could result in growth defects and lipid-related phenotypes.

## Discussion

In this study, we investigate the impact of SmarcAL1 on cellular lipid metabolism, offering both in vitro and in vivo evidence that validate its significance in maintaining cellular lipid homeostasis. Our findings unveil the translocation of SmarcAL1 from nuclei to cytoplasmic peroxisomes, a dynamic process that likely imparts control over cellular lipid metabolism through SmarcAL1-mediated regulation of lipid gene expression. We confirm this by showing that SmarcAL1 gene inactivation reduced the expression of numerous genes responsible for lipid metabolism. Our results highlight the role of SmarcAL1 in regulating lipid catalytic processes and suggest that genetic variations at the SmarcAL1 locus may underlie the observed lipid phenotypes in SIOD patients.

The discovery of SmarcAL1’s involvement in regulating cellular lipid metabolism aligns with the hyperlipidemia observed in SIOD patients^5,8,9^. Our study has revealed that this regulation is likely dependent on its activities in influencing gene expression. The reduced expression of two key genes, LRP1 and LDLR, resulting from SmarcAL1 deficiency, is consistent with the elevated levels of TG and TC, respectively, observed in SIOD patients^8,9^. However, SmarcAL1 KO mice only displayed elevated TG levels without showing apparent changes in TC and LDL-C levels (Fig. S4B–F). This discrepancy may be attributed to the different lipid metabolisms between humans and mice. In humans, mutations in LDLR gene are common causes of hypercholesterolemia^31^. Conversely, in mice LDLR deletion only slightly increases blood cholesterol levels because mice have alternative pathways, such as apolipoprotein E (ApoE) -mediated cholesterol clearance, for regulating cholesterol metabolism^26,27^. Consequently, we suggest that testing SmarcAL1 KO mice on an ApoE KO background would offer deeper insights into the changes in cholesterol levels and provide a more comprehensive understanding of SmarcAL1’s influence on lipid metabolism.

Genetic deficiency of SmarcAL1 in mice and fruit flies was insufficient to induce SIOD phenotypes^16^. In line with this, the SmarcAL1 KO mice in this study did not display obvious developmental or growth defects, such as body weight (Fig. S4A), over a 24-month period. It was proposed that SmarcAL1-regulated gene expression plays a major role in the SIOD development. SmarcAL1 has been shown to be co-localized at the transcriptionally active chromatin regions^16^. However, it is important to note that SmarcAL1 does not conform to the typical definition of a transcription factor, as it lacks sequence-binding specificity^10,36^. Instead, its transcription regulation resides in its core nuclear activity in chromatin remodeling, the details of which remain largely unexplored. Interestingly, both SIOD patients and SmarcAL1-deficient flies and mice, demonstrate higher susceptibility to heat stress, a phenotype related to abnormal cellular metabolic processes resulting from gene expression changes induced by SmarcAL1 deficiency^16^. Consistent with this hypothesis, the findings from this study suggest that SmarcAL1 is important for cellular FA metabolism because its transcriptional regulation is required for the expression of genes associated with peroxisome activity and FA catalytic pathways. Furthermore, according to the tissue-specific expression data (Fig. 5A)^33^, SMARCAL1 is highly expressed in many tissues, including skeletal and cardiac muscles, as well as adipose tissues. Therefore, SmarcAL1 could potentially serve as a key transcription regulator for energy metabolism, a function that is integral to growth and development.

The translocation of SmarcAL1 from nucleus to peroxisomes is an intriguing discovery. Previous reports had suggested the presence of SmarcAL1 in cytoplasm^15^. However, the peroxisomal enrichment of SmarcAL1, as revealed in this study, suggests that it may have additional functions in cytoplasm. Peroxisomes are well-known as pivotal oxidative organelles with essential roles in lipid metabolism. Notably, they participate in the catabolism of FAs, wherein very long-chain FAs are trimmed into medium-chain FAs via beta-oxidation. Subsequently, these medium-chain FAs are oxidized in mitochondria for ATP production^37^. The functional significance of this peroxisomal enrichment of SmarcAL1 remains a subject of further investigation. Alternatively, the peroxisomal enrichment of SmarcAL1 might function as a reservoir, allowing it to translocate back to nucleus when required for transcriptional regulation. Such a mechanism is not uncommon in lipid metabolism. For example, the transcription factor, Sterol Regulatory Element Binding Proteins 1, is usually sequestered in cytoplasm by binding to the endoplasmic reticulum membrane. It can be released to nucleus for transcription activation by sterol-regulated proteolysis when cholesterol synthesis is required^38^. Importantly, the interaction between SmarcAL1 and Angptl3 occurs in cytoplasm, suggesting an additional layer of regulatory control over SmarcAL1 activity. We anticipate that this dynamic shuttling of SmarcAL1 could play an indispensable role in regulating FA metabolism in response to TG storage and expenditure. Further studies would be needed to address these questions.

While our study has provided some insights into the role of SMARCAL1 in lipid metabolism, it is important to acknowledge certain limitations. Our SmarcAL1 KO mouse model did not fully replicate the lipid phenotypes and growth defects observed in humans. This implies the existence of species-specific physiological differences, particularly in lipid metabolism, which should be considered when interpreting our findings. Our research primarily focused on the impact of SmarcAL1 deficiency on lipid metabolism. Lipid metabolism is a complex process influenced by many other biological processes and pathways. Future research should consider broader genetic and molecular analysis, potentially using diverse mouse models. To better understand the clinical relevance of our findings, it is important to investigate the role of SMARCAL1 in human lipid metabolism. This could encompass a range of approaches, including analyzing patient data, conducting genetic association studies, or using patient-derived cell models. Moreover, further efforts should be directed towards unraveling the precise mechanisms through which SmarcAL1 exerts its regulatory influence on lipid metabolism, particularly its role in modulating peroxisome function related to the FA catalytic process. This mechanistic insight will be pivotal in comprehending the finer details of SmarcAL1’s involvement in lipid homeostasis.

## Materials and methods

### Antibodies and biological reagents

All antibodies and reagents used in this study are listed in Supplementary Table 1.

### Cell culture, plasmid constructs and transfections

McA and Huh7 cells, as well as the cell lines generated from this study, were cultured in DMEM medium (Invitrogen) supplemented with 10% FBS (Sigma). Human primary hepatocytes were purchased from Invitrogen/Thermo Fisher. Recovery and culture of the hepatocytes were carried out exactly according to the manufacturer’s procedures.

Fc and Fc-Angptl3 expression constructs were generated by cloning cDNA of Fc and Fc-Angptl3 into pCI-neo mammalian expression vector (Invitrogen) at XhoI-XbaI and NheI-XhoI-NotI sites, respectively. Fc-Angptl4 and Fc-Angptl8 constructs were generated by replacing the cDNA of Angptl3 with those of human Angptl4 and Angptl8 at XhoI-NotI sites based on Fc-Angptl3 expression construct. All the constructs were confirmed by DNA sequencing.

Transfections were performed by using lipofectamine 2000 (Invitrogen) according to the manufacturer’s protocol. The stable Fc and Fc-Angptl3 McA cell lines were obtained by treating the transfected cells with neomycin for selection. For stable Fc/Fc-Angptl3 cells, single clones were obtained by growing single cells in 96-well plates followed by screening with Western blotting analyses.

### *SmarcAL1* gene deletion in Huh7 and McA cells using CRISPR/Cas9 genome editing

All the single guide RNAs (sgRNAs) described in this study were designed using the online tools (http://primedesign.pinellolab.org). The sgRNAs selected have highest quality scores and specificity among all possible candidates. All the sgRNA constructs were confirmed by DNA sequencing.

Generation of heterologous *SmarcAL1* deletion and control McA cells. Two sgRNA oligo fragments (5′-GCGGAGCCTTCAAAGCCAGA-3′ and 5′-GATCGCTTCCGGGTAAAGAT-3′) that target exon 2 of rat *SmarcAL1* gene and a scramble control sgRNA (5′-GCACTACCAGAGCTAACTCA-3′) were subcloned into pSpCas9(BB)-2A-Puro (PX459) V2.0 vector (addgene)^39^. The constructs were transfected into McA cells. Subsequently, the cells were cultured in presence of puromycin (5 μg/ml) for 5 days. The transfected cells were then subjected for single clone selection. To confirm the *SmarcAL1* gene deletion, PCR reactions with a set of primers (5′-CTTCTGCCAACTTCCCAG-3′ and 5′-GCAGACGCTATGgtaatgag-3′) that cover the target sequences, were carried out using the genomic DNA purified from the single clones as templates. The PCR products were used for T7 endonuclease I (New England Biolabs) assay^40^. The expression of SmarcAL1 protein was confirmed by Western analysis with anti-SmarcAL1 and -β-actin antibodies (Fig. 2A, bottom).

Generation of homologous *SMARCAL1* and control Huh7 cells. *SMARCAL1* KO and control cells were generated as previously reported^41^. Three sgRNA oligoes (5′-GATTGAAGAGAATCGACAAA-3′, 5′-AGCTCGGGCACCTCCATTGC-3′, and 5′-AGGGAGTCTTGTAAGCCAGT-3′) that target exon 2 of human *SMARCAL1* gene and the scramble control sgRNA as above were subcloned into lentiGuide-Puro vector (addgene). Briefly, the sgRNA oligoes were first subcloned into lentiGuide-Puro vector. The vector constructs together with accessory plasmids for lentivirus assembly were subsequently co-transfected into HEK293T cells for lentivirus production^42,43^. Packaged viruses were used to transduce the Cas9-expressing Huh7 cells for ~16 h. The transfected cells were treated with puromycin (5 μg/ml) for five days. Subsequently, single cell clones were screened, and the *SMARCAL1* KO was confirmed with Western analysis with anti-SmarcAL1 and -β-actin antibodies (Fig. 2B, bottom).

### Mice treatment and handling

WT mice (C57BL/6 J) were obtained from Jackson Laboratory. *SmarcAL1* KO mice were generated from C57BL/6 J mice at the Genome Modification Facility of Harvard University according to the procedures reported previously^44^. Briefly, a total of 4 sgRNA oligoes targeting exon 2 of mouse SmarcAL1 gene were synthesized (Invitrogen). Validation of these sgRNAs was carried out as previously reported^45^. Briefly, the oligoes were subcloned into pX602 plasmid containing Cas9 from *Staphylococcus aureus* (SaCas9). A genomic fragment (767 bp) containing the target sites of the sgRNAs from mouse SmarcAL1 gene was subcloned into pCAG-EGxxFP plasmids. The two plasmid constructs were co-transfected into HEK293T cells using lipofectamine 2000 (Invitrogen). The EGFP fluorescence intensity representing the cleavage efficiency of the sgRNAs was monitored under a fluorescent microscope after 48 h of transfection.

Two sgRNA sequences with the strongest activity, 5′-CGGCCCGTCCCAGTCCAAGC-3′ and 5′-TTGGGTTATAAATCCAGCGA-3′, were used for synthesis of two oligoes containing T7 minimum promoter element (lower case) at the upstream of the sgRNA sequences (5′-ttaatacgactcactatagg CGGCCCGTCCCAGTCCAAGC-3′ and 5′-ttaatacgactcactataggTTGGG TTATAAATCCAGCGA-3′) (see MEGAshortscript protocol, Ambion/Life Technologies). The annealed sense and antisense oligoes were then used as templates for in vitro transcription using MEGAshortscript Kit according to the manufacturer’s procedures. RNA was purified using MEGAclear Kit (Ambion/Life Technologies). The purified RNAs were further extracted with phenol/chloroform and precipitated with ethanol. RNA precipitates were washed with 70% ethanol 4 times. The pure sgRNAs and Cas9 protein (PNA Bio) were used for injection into the mouse zygotes.

A total of 37 mice (19 males and 18 females) were obtained from F0 generation. Genotypes were screened using PCR with the genomic DNA from mouse tails and a pair of primers from mouse SmarcAL1 gene locus (sense, 5′-CTTTTACCAACTTCCCAACTA-3′, and antisense, 5′-CATAGTGTCTGCTGGGCAGAG-AC-3′). After screening, 23 were found to carry heterologous (HET) *SmarcAL1* gene deletions. The confirmed mice (9 males and 9 females) carrying the *SmarcAL1* gene deletion were used for breeding. The F0 HET mice were used to mate with C57BL/6 J mice to generate F1 HET mice. F1 HET mice were bred with F1 HET to generate homozygous *SmarcAL1* KO mice. After several rounds of breeding, we obtained the homologous *SmarcAL1* KO mice confirmed by genetic analysis and Western analysis for null *SmarcAL1* protein expression in the livers (Fig. 2C). The confirmed HET and KO mice were bred for subsequent lineage expanding, in which the KO mice were backcrossed with WT mice (C57BL/6 J). The HET mice were bred to generate WT, HET and KO mice that were used for the analyses as indicated in Fig. 2D–F. For all the analyses, comparisons were made among WT, HET, and KO littermates. All mice were fed with a standard chow diet and maintained on a 12 h light/12 h dark cycle. All procedures used in animal studies were approved by the pertinent Institutional Animal Care and Use Committee (IACUC) and were consistent with local, state, and federal regulations as applicable.

To minimize the blood TG variations, mice were synchronized in fasting and refeeding as previously reported^46^. Briefly, mice were trained with fasting for 12 h followed by refeeding for 12 h for 3 days before experiments. Blood samples were collected at the end of the fasting period.

Mouse blood samples were collected from retro-orbital sinus after anesthesia procedure. Blood was spined immediately for plasma preparation. In some experiments, mice were sacrificed. The liver samples were collected.

### Cell and liver extracts and Western blotting analysis

Whole cell extracts were prepared by solubilizing cells in the lysis buffer containing 150 mM NaCl, 50 mM Tris (pH 7.5), 1% IGPAL-CA-630 (Sigma # I8896) and protease inhibitor cocktail (Roche) and rotating at 4 °C for 15 min followed by centrifugation at 13,000 rpm for 10 min.

Liver lysates were prepared by manual slicing of livers and solubilizing in 1X Tris Buffered Saline (TBS, pH 7.4) containing 1% NP-40, 0.1% sodium deoxycholate and complete protease inhibitor cocktail (Roche) followed by rotating at 4 °C for 1 h and spinning at 13,000 rpm for 20 min. Protein was quantified by Pierce BCA (bicinchoninic acid) protein assay (Thermo Fisher). The liver lysate (~50 μg protein) was used for Western analysis.

Western blotting analysis was performed using precast Mini-PROTEAN TGX SDS-PAGE (4–20%) (Bio-Rad). Blots were probed with the primary antibodies as indicated in figures and appropriate secondary antibodies.

### Affinity PD assay and recombinant protein purification

Fc and Fc-Angptl3 affinity PD assays were performed as previously described^20,21^. Briefly, protein-A beads were washed with the lysis buffer, then the washed beads were incubated with the cell extracts as described above rotating at 4 °C for ~12 h. The mixtures were spined at 2000 rpm for 5 min. The protein-bound beads were washed three times with the lysis buffer. The precipitates were loaded on precast Mini-PROTEAN TGX SDS-PAGE (4–20%) for Western analysis.

### RNA extraction, microarray and RNA-seq transcriptome-wide analyses

Total RNA was purified using RNeasy Mini Kit (Qiagen) according to the manufacturer’s procedures. All purified RNA samples were subjected to Bioanalyzer trace analysis (Agilent) for RNA quality control. All RNA samples (each sample with three replicates) used for transcriptome analyses passed optimum specifications for total RNAs (RIN score of >7.0, 260/280 = 1.8–2.0 and 260/230 = 1.8–2.0 as key markers of RNA purity). RNA-seq analyses were performed at the Mass General Brigham Biobank Genomics Core. For RNA-seq analyses, libraries were prepared using TruSeq® Stranded Total RNA Library Prep Human/Mouse/Rat (20020597, Illumina). Sequencing reads with 75 bp paired ends were generated on an Illumina HiSeq 2500 platform according to manufacturer’s supplied protocol. Raw reads were processed, and trimmed reads were aligned to the human genome (hg19) or rat genome (Rattus_norvegicus.Rnor_6.0.85.chr.gtf) using STAR aligner (2.4.2). All further DE analysis was performed using cuffdiff in cufflinks (2.2.1). Differential genes with p and q values less than 0.05 were used for GO enrichment analysis using DAVID (the database for annotation, visualization and integrated discovery)^47^. The pathways and biological processes with Bonferroni, Benjamini, and FDR less than 0.05 are considered statistically significant.

### Imaging analyses

All the phase images were taken using regular light microscope. Immunofluorescence analysis was done essentially as previous described^48^. Briefly, cells were fixed with a PBS buffer containing 3% paraformaldehyde (Sigma, P6148) and 2% sucrose for 10 min and permeabilized with Triton X-100 buffer (0.5% Triton X-100, 20 mM HEPES-KOH, pH 7.9, 50 mM NaCl, 3 mM MgCl2, 300 mM sucrose). The cells were then blocked in PBS buffer containing 0.5% bovine serum albumin and 0.2% gelatin (Sigma G7765) and incubated with primary antibodies and appropriate secondary antibodies. The coverslips were mounted in mounting media (Vector Laboratories, P36931). All the images were collected by scanning the slides with Leica SP5 AOBS Scanning Laser Confocal Microscope. The images were analyzed with CellProfiler or otherwise described in individual figures. Constant thresholds were applied for all the images for the relevant series of experiments. Particle analysis was used to collect the cell particle number and intensity data.

### Measurements of mouse plasma lipids and lipoproteins, and Western analysis of liver extracts

Mouse plasma TG was measured with triglyceride colorimetric assay kit (Cayman, 10010303) according to the manufacturer’s protocol. Pooled plasma was subjected to fast phase liquid chromatography (FPLC) gel filtration (Cytiva) using two serial Superose 6 increase columns (Cytiva) (performed at Dr. Rader’s lab). Triglyceride and cholesterol colorimetric plate assays were performed on FPLC fractions using Infinity Triglyceride Reagent (for triglyceride) and Infinity Cholesterol Reagent (for cholesterol), respectively, (Thermo Scientific).

### Dil-VLDL uptake assays and nascent ApoB-100 secretion and measurement

For VLDL/LDL uptake assays, cells were incubated in serum-free medium (2 h) followed by incubation in serum-free medium containing Dil-VLDL^49^ (5 μg/ml, Invitrogen) for 1 h. The cells were then fixed, co-stained with DAPI, and analyzed under confocal microscope as described in imaging analyses.

Time-course ApoB-100 secretion assays were carried out as previously reported^41^. Briefly, *SMARCAL1* KO and control Huh7 cells (3 clones for each) were splitted into 6-well plates with replicates for testing and cell counting. Next day, the cells were washed with serum-free media and incubated with fresh serum-free media. At various times, media samples were collected and measured for ApoB-100 level using ELISA kit (MABTECH) according to the manufacturer’s instructions. The levels of ApoB-100 were normalized with cell numbers.

### SILAC cell culture, affinity PD and mass spectrometry analysis

The SILAC analysis was performed at the Proteomics Platform, Broad Institute of MIT and Harvard. The procedures for SILAC media preparation and cell culture conditions were adapted from the standard steps as previously described^18,50–52^. Briefly, base media was prepared by addition of L-methionine and 200 mg/L of L-Proline according to the standard formulations for DMEM (Caisson Labs). This base media was used for preparation of SILAC light and heavy labeling media with addition of l-arginine (Arg0) and l-lysine (Lys0) (light), ^13^C_6_^15^N_4_-l-arginine (Arg10) and ^13^C_6_^15^N_2_-l-Lysine (Lys8) (heavy), respectively. Both media were supplemented with the full complement of amino acids at the standard concentration, 2 mM L-glutamine (GIBCO), 10% dialyzed FBS (Sigma) and antibiotics (GIBCO). Fc and Fc-Angptl3 McA cells were grown in the light and heavy labeling media, respectively, under standard conditions. Cells were continuously grown with at least eight cell divisions in the labeling media.

At the end of labeling, cells were washed twice with ice cold PBS. Equal number of Fc and Fc-Angptl3 cells were used for lysate preparation with the lysis buffer containing 50 mM Tris-HCl (pH 7.8), 150 mM NaCl, 1% NP-40, 0.1% sodium deoxycholate, 1 mM EDTA and complete protease inhibitor cocktail (Roche). The cell lysates were rotated at 4 °C for 15 min and centrifuged at 13,000 rpm for 20 min. The protein concentrations of the supernatants were quantified using Bradford assay. About 4 mg extracts from Fc and Fc-Angptl3 cells were incubated with protein-A beads (pre-washed 2 times in PBS and one time in the lysis buffer) with rotation at 4 °C for overnight. The mixtures were gently spined at 2000 rpm for 5 min. The pellets were washed three times with the lysis buffer as above and one time in the same buffer without NP40. The protein-bound beads were subsequently used for mass spectrometric analysis as described below.

The above beads were treated in 2 mM DTT for 30 min at RT and then alkylated with 10 mM iodoacetamide for 45 min at dark. The proteins were eluted with 4XLDS buffer (Invitrogen) and heated at 70 °C for 10 min. The eluted proteins were separated on a gradient Bis-Tris gel (4–12%) (Nupage, Invitrogen) followed by Coomassie staining (Simply Blue, Invitrogen). Each gel lane was cut into small pieces, which were first destained in a buffer containing 50% ethanol and 50 mM ammonium bicarbonate, then dehydrated in 100% ethanol. Enzymatic digestion was performed by adding sufficient trypsin (12.5 ng/μL) with overnight shaking. The samples were then quenched with 100 μL of 1% trifluoroacetic acid (TFA) and the peptides from each gel slice were captured on C18 stage tips (Thermo Fisher Pierce). Captured peptides were recovered in 50 μL of 80% acetonitrile/0.1% TFA. Organic solvents were removed by drying the mixtures in an evaporative centrifuge.

The recovered peptides were resolved with 3% acetone (ACN) in 0.1% formic acid (FA). The mixtures were subsequently separated on an online nanoflow EASY-nLC 1000 UHPLC system (Thermo Fisher Scientific) and analyzed on a benchtop Orbitrap Q Exactive plus mass spectrometer (Thermo Fisher Scientific). The peptide samples were injected onto a capillary column (Picofrit with 10 μm tip opening/75 μm diameter, New Objective, PF360-75-10-N-5) packed in-house with 20 cm C18 silica material (1.9 μm ReproSil-Pur C18-AQ medium, Dr. Maisch GmbH, r119.aq). The UHPLC setup was connected with a custom-fit microadapting tee (360 μm, IDEX Health & Science, UH-753), and capillary columns were heated to 50 °C in column heater sleeves (Phoenix-ST) to reduce backpressure during UHPLC separation. Injected peptides were separated at a flow rate of 200 nL/min with a linear 50 min gradient from 100% solvent A (3% ACN, 0.1% FA) to 30% solvent B (90% ACN, 0.1% FA), followed by a linear 9 min gradient from 30% solvent B to 60% solvent B and a 1 min ramp to 90% solvent B. Data-dependent acquisition was obtained using Xcalibur 2.2 software in positive ion mode at a spray voltage of 2.00 kV. Each sample was run for 150 min, including sample loading and column equilibration times. MS1 Spectra were measured with a resolution of 60,000, an AGC target of 3 × 10^6^ and a mass range from 300 to 1800 *m/z*. Up to 12 MS2 spectra per duty cycle were triggered at a resolution of 15,000, an AGC target of 5 × 10^4^, an isolation window of 1.5 *m/z* and a normalized collision energy of 25. Peptides that triggered MS2 scans were dynamically excluded from further MS2 scans for 20 s.

All mass spectra were analyzed with MaxQuant software version 1.3.0.5. For peptide identification MS/MS spectra were searched against rat Uniprot database to which a set of common laboratory contaminant proteins were appended as well as Rat Angptl3 with and without TAG. Search parameters included: Trypsin/P was selected as the digestion enzyme, a maximum of 3 labeled amino acids and 2 missed cleavages per peptide were allowed. Fragment ion mass tolerance was set to 20 ppm. The mass tolerance for precursor ions was set to 20 ppm for the first search (used for nonlinear mass re-calibration) and 6 ppm for the main search. Allowed variable modifications were oxidation of methionine, protein N-terminal acetylation as variable modifications. Carbamidomethylation was used as fixed modification. For identification we applied a maximum FDR of 1% separately on protein, peptide, and PTM-site level. We required 2 or more unique/razor peptides for protein identification and a ratio count of 2 or more for protein quantification per replicate measurement. To assign interacting proteins, we used the Limma package in the R environment to calculate moderated *t* test *p* values^53^.

### Total cell lipid and polar lipid extract preparation, and metabolite mass spectrometry analysis

The metabolomics analysis was performed at the Metabolomics Program, Broad Institute of MIT and Harvard. Preparation of total cell lipid and polar lipid extracts was carried out as previously described^54^. Briefly, for lipid extract, cells were washed with cold PBS (no Mg^2+^/no Ca^2+^) and immediately 800 µL of ice-cold isopropanol (HPLC grade) was added. The cells were then scraped and transferred to a 1.5 mL tube and kept at 4 °C for 1 h. For polar lipid extract, after wash, ~800 µL of −80 °C 80% methanol (LC/MS grade) was added to cells, which were then transferred to −80 °C for 15 min. Cells were scraped and collected to a 1.5 mL tube. For both extracts, cell suspension was vortexed and cell debris was removed by spinning at 9000 × *g* at 4 °C for 10 min. Supernatant was used for the metabolite mass spectrometry analysis as previously described^54^.

### Genetic association analysis

The genetic results used in this study were primarily obtained from Association to Function Knowledge Portal (date of access: March 2023; https://a2f.org/). The results were generated through a meta-analysis of genetic association studies, utilizing a bottom-line integrative analysis approach^34^. This method involves pooling data from multiple studies with similar designs and sampling to estimate the effect of a genetic variant on a phenotype. This approach increases the power to detect novel significant associations that may not be detectable at the level of individual datasets but become significant when multiple studies are included.

In this study, the bottom-line variant associations for multiple phenotypes of interest were focused on the SMARCAL1 gene locus, which is located between 217,177,137 and 217,447,776 on chromosome 2 according to the hg19 (GRCh37) genome build. Details about the genetic data related to this study and documentations about the bottom-line integrative analysis can be available at the Association to Function Knowledge portal (https://a2f.org/).

To visualize the regional associations, association plots were generated using R, as previously reported^55^. This method allowed for a closer examination of the genetic associations in the SMARCAL1 gene locus for multiple phenotypes of interest.

### Statistics and reproducibility

Statistic comparisons of groups including the student’s *t* test were performed using Prism 9 (GraphPad). Two-way analysis of variance (ANOVA) was performed by comparing the matched means of three replicates from experimental groups with those from the control group as indicated in figures. Detailed group comparisons and sample numbers were provided in individual figure and Supplementary Information and Data. In general, *p* ≤ 0.05 were considered statistically significant (*p < 0.05; **p < 0.01; ***p < 0.001; and ****p < 0.0001).

### Reporting summary

Further information on research design is available in the Nature Portfolio Reporting Summary linked to this article.

### Supplementary information

Supplementary Information

Description of Additional Supplementary Files

Supplementary Data 1

Supplementary Data 2

Supplementary Data 3

Supplementary Data 4

Supplementary Data 5

Supplementary Data 6

Reporting Summary

## Data Availability

The original blot images of Figs. 1D, 2A–C and S1 are provided in specific Supplementary Figs. S8–12. The source data used in Figs. 4C and 5B, and Supplementary Figs. 2, 3, 6 and 7 are provided in the Supplementary Datasets 1–6. The RNA-seq data presented in this paper were deposited in Gene Expression Omnibus (GEO) (https://www.ncbi.nlm.nih.gov/geo/). The accession number of the RNA-seq data from the McA cells with heterologous SmarcAL1 KO is GSE221373. The original mass spectra have been deposited in the public proteomics repository MassIVE (http://massive.ucsd.edu) using the identifier: MSV000085620. The data is accessible via ftp://MSV000085620@massive.ucsd.edu.

## References

[1] Coleman MA, Eisen JA, Mohrenweiser HW (2000). Cloning and characterization of HARP/SMARCAL1: a prokaryotic HepA-related SNF2 helicase protein from human and mouse. Genomics.

[2] Ryan DP, Owen-Hughes T (2011). Snf2-family proteins: chromatin remodellers for any occasion. Curr. Opin. Chem. Biol..

[3] Elizondo LI (2006). Schimke immuno-osseous dysplasia: a cell autonomous disorder?. Am. J. Med. Genet. A.

[4] Boerkoel CF (2002). Mutant chromatin remodeling protein SMARCAL1 causes Schimke immuno-osseous dysplasia. Nat. Genet.

[5] Clewing JM (2007). Schimke immuno-osseous dysplasia: a clinicopathological correlation. J. Med. Genet.

[6] Morimoto M (2012). Reduced elastogenesis: a clue to the arteriosclerosis and emphysematous changes in Schimke immuno-osseous dysplasia?. Orphanet J. Rare Dis..

[7] Boerkoel CF (2000). Manifestations and treatment of Schimke immuno-osseous dysplasia: 14 new cases and a review of the literature. Eur. J. Pediatr..

[8] Lucke T (2004). Generalized atherosclerosis sparing the transplanted kidney in Schimke disease. Pediatr. Nephrol..

[9] Spranger J (1991). Schimke immuno-osseous dysplasia: a newly recognized multisystem disease. J. Pediatr..

[10] Medvedeva YA (2015). EpiFactors: a comprehensive database of human epigenetic factors and complexes. Database (Oxf.).

[11] Zhang P, Torres K, Liu X, Liu CG, Pollock RE (2016). An overview of chromatin-regulating proteins in cells. Curr. Protein Pept. Sci..

[12] Ciccia A (2009). The SIOD disorder protein SMARCAL1 is an RPA-interacting protein involved in replication fork restart. Genes Dev..

[13] Driscoll R, Cimprich KA (2009). HARPing on about the DNA damage response during replication. Genes Dev..

[14] Patne K (2017). BRG1 and SMARCAL1 transcriptionally co-regulate DROSHA, DGCR8 and DICER in response to doxorubicin-induced DNA damage. Biochim Biophys. Acta Gene Regul. Mech..

[15] Haokip DT (2016). Transcriptional regulation of Atp-dependent chromatin remodeling factors: Smarcal1 and Brg1 mutually co-regulate each other. Sci. Rep..

[16] Baradaran-Heravi A (2012). Penetrance of biallelic SMARCAL1 mutations is associated with environmental and genetic disturbances of gene expression. Hum. Mol. Genet.

[17] Koishi R (2002). Angptl3 regulates lipid metabolism in mice. Nat. Genet..

[18] Kronke J (2014). Lenalidomide causes selective degradation of IKZF1 and IKZF3 in multiple myeloma cells. Science.

[19] Wen Q (2012). Identification of regulators of polyploidization presents therapeutic targets for treatment of AMKL. Cell.

[20] Angata T, Varki A (2000). Cloning, characterization, and phylogenetic analysis of siglec-9, a new member of the CD33-related group of siglecs. Evidence for co-evolution with sialic acid synthesis pathways. J. Biol. Chem..

[21] Xu YX (2015). The glycosylation-dependent interaction of perlecan core protein with LDL: implications for atherosclerosis. J. Lipid Res..

[22] Yoshida K, Shimizugawa T, Ono M, Furukawa H (2002). Angiopoietin-like protein 4 is a potent hyperlipidemia-inducing factor in mice and inhibitor of lipoprotein lipase. J. Lipid Res..

[23] Quagliarini F (2012). Atypical angiopoietin-like protein that regulates ANGPTL3. Proc. Natl Acad. Sci. USA.

[24] Chi X (2017). ANGPTL8 promotes the ability of ANGPTL3 to bind and inhibit lipoprotein lipase. Mol. Metab..

[25] Cho SW, Kim S, Kim JM, Kim JS (2013). Targeted genome engineering in human cells with the Cas9 RNA-guided endonuclease. Nat. Biotechnol..

[26] Powell-Braxton L (1998). A mouse model of human familial hypercholesterolemia: markedly elevated low density lipoprotein cholesterol levels and severe atherosclerosis on a low-fat chow diet. Nat. Med..

[27] Marais AD (2019). Apolipoprotein E in lipoprotein metabolism, health and cardiovascular disease. Pathology.

[28] Kashiwayama Y (2005). Role of Pex19p in the targeting of PMP70 to peroxisome. Biochim Biophys. Acta.

[29] Lillis AP, Van Duyn LB, Murphy-Ullrich JE, Strickland DK (2008). LDL receptor-related protein 1: unique tissue-specific functions revealed by selective gene knockout studies. Physiol. Rev..

[30] Xu YX (2020). EDEM3 modulates plasma triglyceride level through its regulation of LRP1 expression. iScience.

[31] Abifadel M, Boileau C (2023). Genetic and molecular architecture of familial hypercholesterolemia. J. Intern Med..

[32] Thul, P. J. et al. A subcellular map of the human proteome. *Science* **356**, 10.1126/science.aal3321 (2017). 10.1126/science.aal3321 28495876

[33] Oliva, M. et al. The impact of sex on gene expression across human tissues. *Science* **369**, 10.1126/science.aba3066 (2020). 10.1126/science.aba3066 PMC8136152 32913072

[34] Costanzo MC (2023). The type 2 diabetes knowledge portal: an open access genetic resource dedicated to type 2 diabetes and related traits. Cell Metab..

[35] Pulit SL (2019). Meta-analysis of genome-wide association studies for body fat distribution in 694 649 individuals of European ancestry. Hum. Mol. Genet.

[36] Lambert SA (2018). The human transcription factors. Cell.

[37] Lodhi IJ, Semenkovich CF (2014). Peroxisomes: a nexus for lipid metabolism and cellular signaling. Cell Metab..

[38] Wang X, Sato R, Brown MS, Hua X, Goldstein JL (1994). SREBP-1, a membrane-bound transcription factor released by sterol-regulated proteolysis. Cell.

[39] Ran FA (2013). Genome engineering using the CRISPR-Cas9 system. Nat. Protoc..

[40] Cho SW (2014). Analysis of off-target effects of CRISPR/Cas-derived RNA-guided endonucleases and nickases. Genome Res.

[41] Xu YX (2018). Role of angiopoietin-like 3 (ANGPTL3) in regulating plasma level of low-density lipoprotein cholesterol. Atherosclerosis.

[42] Sanjana NE, Shalem O, Zhang F (2014). Improved vectors and genome-wide libraries for CRISPR screening. Nat. Methods.

[43] Shalem O (2014). Genome-scale CRISPR-Cas9 knockout screening in human cells. Science.

[44] Lin, Y. C. et al. One-step CRISPR/Cas9 method for the rapid generation of human antibody heavy chain knock-in mice. *EMBO J*. **37**, 10.15252/embj.201899243 (2018). 10.15252/embj.201899243 PMC6138433 30087111

[45] Fujihara Y, Ikawa M (2014). CRISPR/Cas9-based genome editing in mice by single plasmid injection. Methods Enzymol..

[46] Banfi S, Gusarova V, Gromada J, Cohen JC, Hobbs HH (2018). Increased thermogenesis by a noncanonical pathway in ANGPTL3/8-deficient mice. Proc. Natl Acad. Sci. USA.

[47] Huang da W, Sherman BT, Lempicki RA (2009). Systematic and integrative analysis of large gene lists using DAVID bioinformatics resources. Nat. Protoc..

[48] Xu YX, Liu L, Caffaro CE, Hirschberg CB (2010). Inhibition of Golgi apparatus glycosylation causes endoplasmic reticulum stress and decreased protein synthesis. J. Biol. Chem..

[49] Stephan ZF, Yurachek EC (1993). Rapid fluorometric assay of LDL receptor activity by DiI-labeled LDL. J. Lipid Res.

[50] Ong SE, Mann M (2006). A practical recipe for stable isotope labeling by amino acids in cell culture (SILAC). Nat. Protoc..

[51] Sandoval GJ (2018). Binding of TMPRSS2-ERG to BAF chromatin remodeling complexes mediates prostate oncogenesis. Mol. Cell.

[52] Xu YX (2020). Interactomics analyses of wild-type and mutant A1CF reveal diverged functions in regulating cellular lipid metabolism. J. Proteome Res.

[53] Ong SE (2009). Identifying the proteins to which small-molecule probes and drugs bind in cells. Proc. Natl Acad. Sci. USA.

[54] Mascanfroni ID (2015). Metabolic control of type 1 regulatory T cell differentiation by AHR and HIF1-alpha. Nat. Med.

[55] Diabetes Genetics Initiative of Broad Institute of, H. (2007). Genome-wide association analysis identifies loci for type 2 diabetes and triglyceride levels. Science.

