# Supplementary material for: Chromatin regulator SMARCAL1 modulates cellular lipid metabolism
Source: Commun Biol. 2023 Dec 21;6:1298. doi: 10.1038/s42003-023-05665-6 (PMC10739977; doi:10.1038/s42003-023-05665-6)
Supplement: Supplementary file 1 — Supplementary Information [file 42003_2023_5665_MOESM1_ESM.pdf]

# Supplementary Information

## Supplementary Information includes:

Supplementary Figures S1-S7.

Supplementary Figures S8-S12, the original blot images of Figures 1D, 2A-C and S1.

Supplementary Table 1, Materials used in this study.

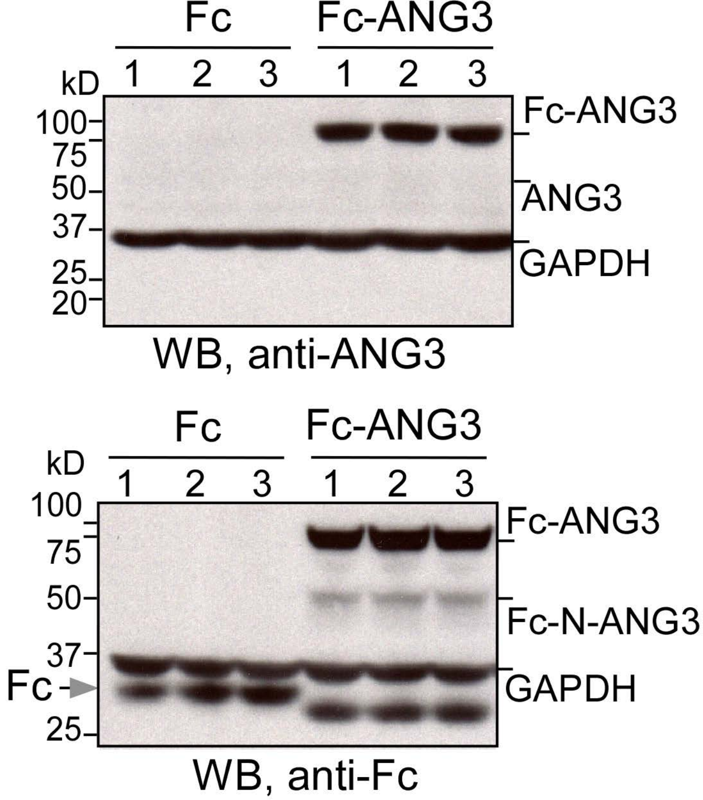


**Fig. S1**. **Expression analysis of the stable McA cell lines carrying Fc or Fc-Angptl3 expressing constructs**. Western analysis of Fc and Fc-Angptl3 (ANG3) expression in the cell lines (three individual clones for each cell line) with antibodies for human Angptl3 (top), Fc (bottom), and GAPDH. See Figure S8 for the full images of this figure. Fc-N-ANG3, Fc-tagged N-terminal Angptl3.

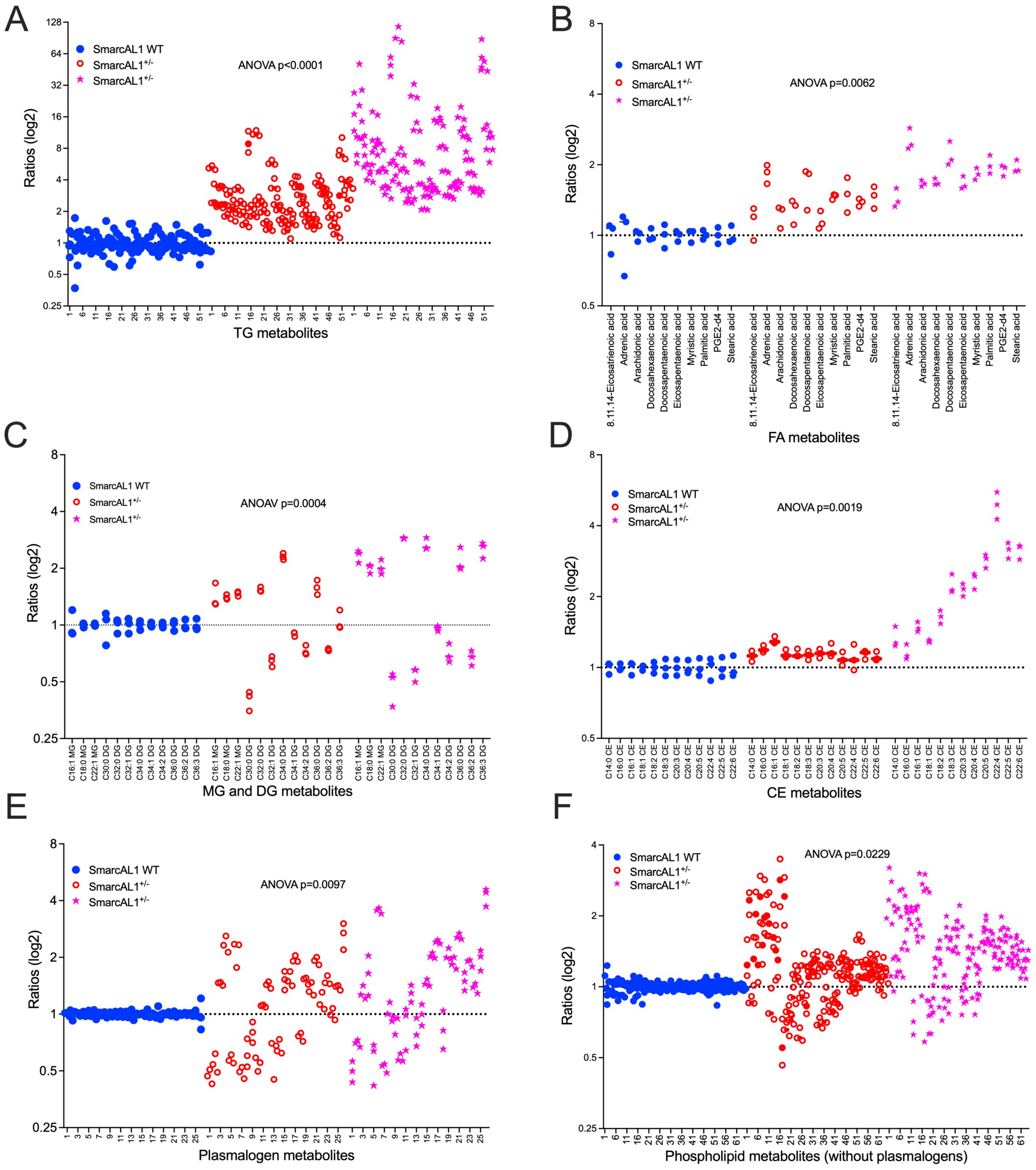


**Fig. S2. Heterologous inactivation of SmarcAL1 gene induces TG and FA accumulation in McA cells**. Total lipid and polar lipid extracts from SmarcAL1$^{+/-}$ and WT control McA cells (three clones for each cell line) were analyzed with mass spectrometry for metabolite changes. Individual metabolites were normalized with cell numbers. Y axis represents the ratios that were calculated by comparing metabolites with the averages of those from three clones of WT control cells. X axis represents the full spectrum of metabolite isoforms from each category (see Supplementary Data 1 for metabolite details). C1 and C4 represent two McA clones with heterologous inactivation of SmarcAL1 gene. P values from two-way ANOVA are indicated.

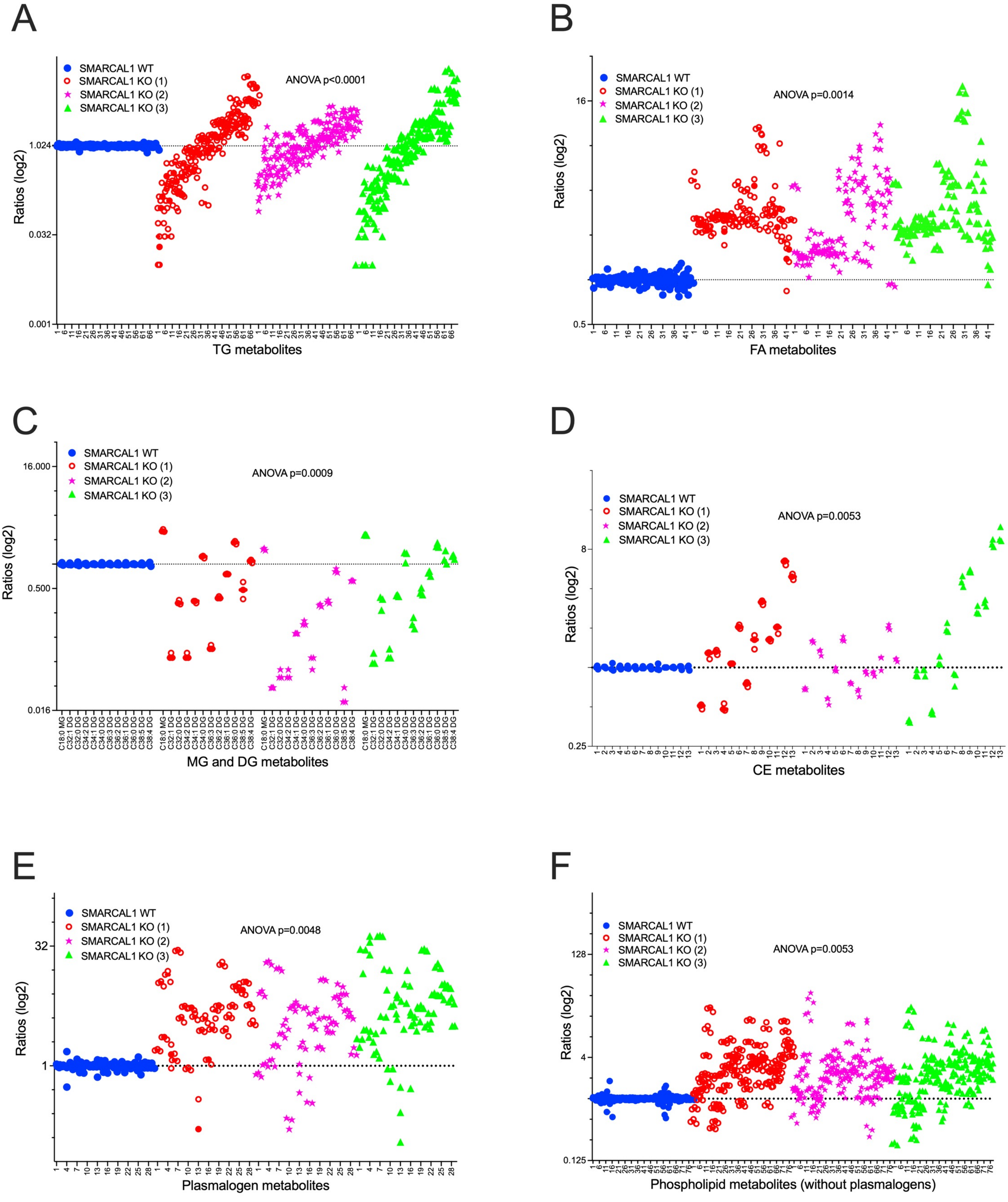


**Fig. S3. Homologous inactivation of SMARCAL1 gene induces TG and FA accumulation in Huh7 cells**. Total lipid and polar lipid extracts from SMARCAL1$^{-/-}$ and WT control Huh7 cells (three clones for each cell line) were analyzed with mass spectrometry for metabolite changes. Individual metabolites were normalized with cell numbers. Y axis represents the ratios that were calculated by comparing metabolites with the averages of those from three clones of WT control cells. X axis represents the full spectrum of metabolite isoforms from each category (see Supplementary Data 2 for metabolite details). P values from two-way ANOVA are indicated.

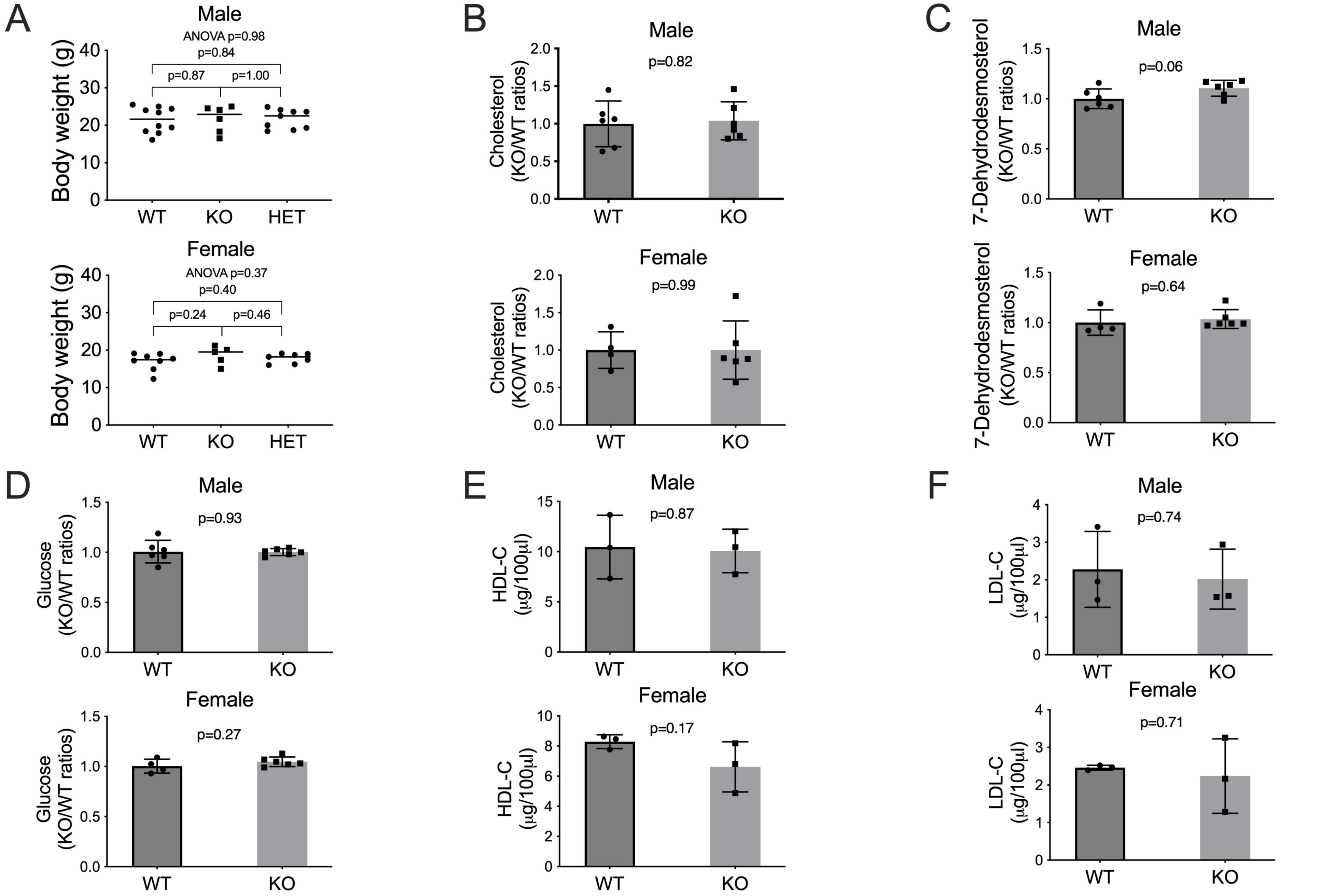


**Fig. S4. Comparative analysis of background data from SmarcAL1 KO mice and WT controls. A.** Body weight comparison. Comparison of body weight among male (top) and female (bottom) littermates of WT (SmarcAL1$^{+/+}$), HET (SmarcAL1$^{+/-}$), and KO (SmarcAL1$^{-/-}$) mice at age of 5-8 weeks. Each dot represents a single mouse. **B** to **D**. Total cholesterol (TC), 7-Dehydrodesmosterol (a cholesterol intermediate), and glucose levels. Comparison of TC (**B**), 7-Dehydrodesmosterol (**C**) and glucose (**D**) in male (top) and female (bottom) WT and KO littermates. The levels were measured using mass spectrometry, and the ratios were calculated by comparing KO mice to WT controls. **E** and **F**. HDL-C and LDL-C levels: Comparison of HDL-C (**E**) and LDL-C (**F**) levels in male (top) and female (bottom) KO and WT littermates. The levels were calculated based on FPLC data (see Fig. 2F). P values were calculated with t test or two-way ANOVA as indicated.

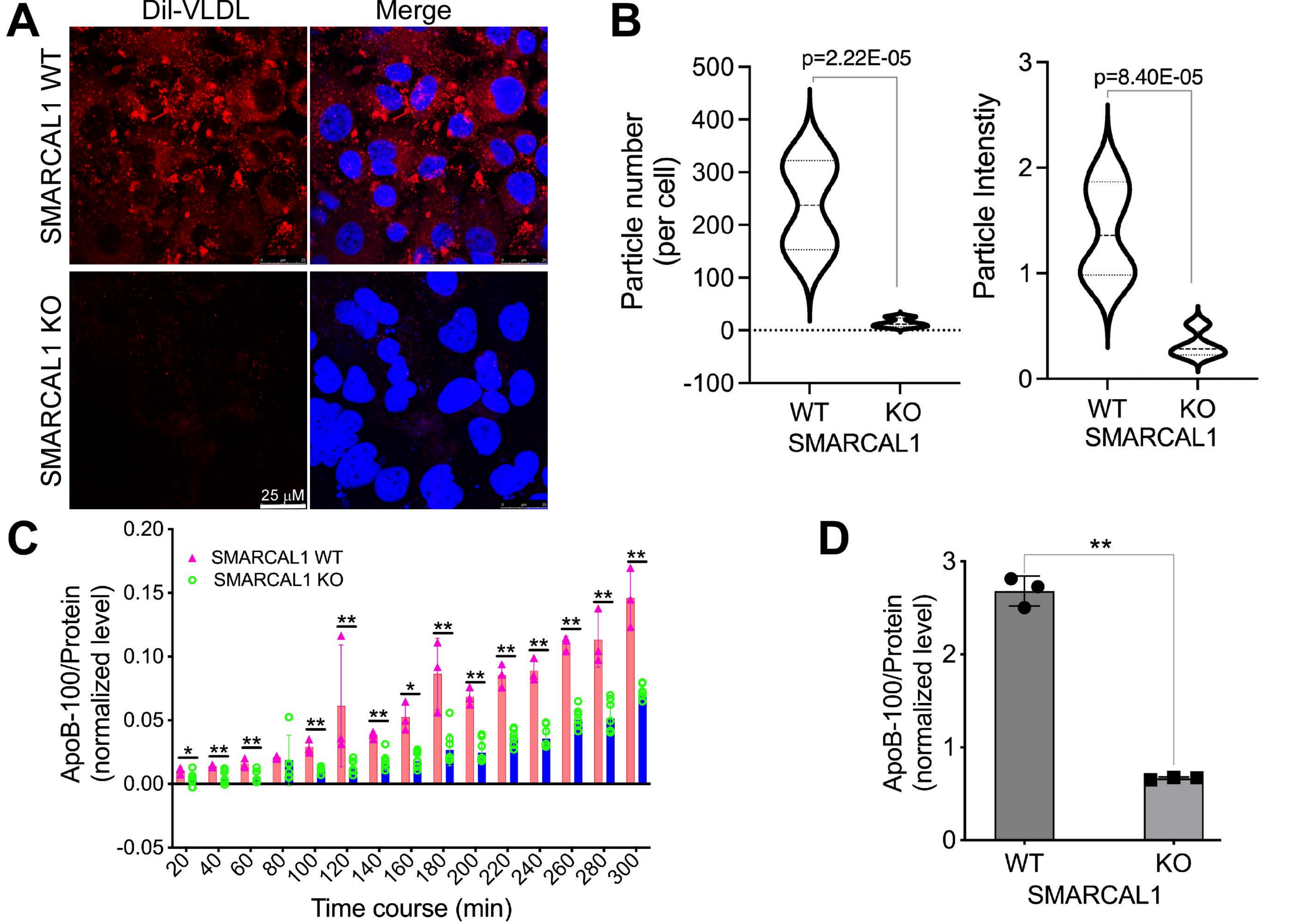


**Fig. S5**. **Inactivation of SMARCAL1 gene blocks very low-density lipoprotein (VLDL) uptake and reduces the secretion of nascent apolipoprotein B-100 (ApoB-100)**. **A**. VLDL uptake analysis. SMARCAL1 KO and WT control Huh7 cells (Three clones for each cell line) were incubated with Dil-VLDL and analyzed by confocal microscope for Dil-VLDL uptake. **B**. Image quantification. Quantification of Dil-VLDL particle numbers and intensity was carried out for the images as in A from three independent experiments. The averages and deviations were calculated from three clones of the KO and control cells (~3,000 cells for each). **C**. and **D**. Nascent ApoB-100 secretion analysis. The ApoB-100 secretion from SMARCAL1 KO and WT control Huh7 cells (Three clones for each cell line. Three replicates for each KO clone) was analyzed in a time course (C) or overnight incubation (D) using ApoB ELISA kit. ApoB-100 protein was normalized with cell numbers. P values calculated from t test by comparing SMARCAL1 KO with the control. *, $P<0.05$; **, $P<0.01$.

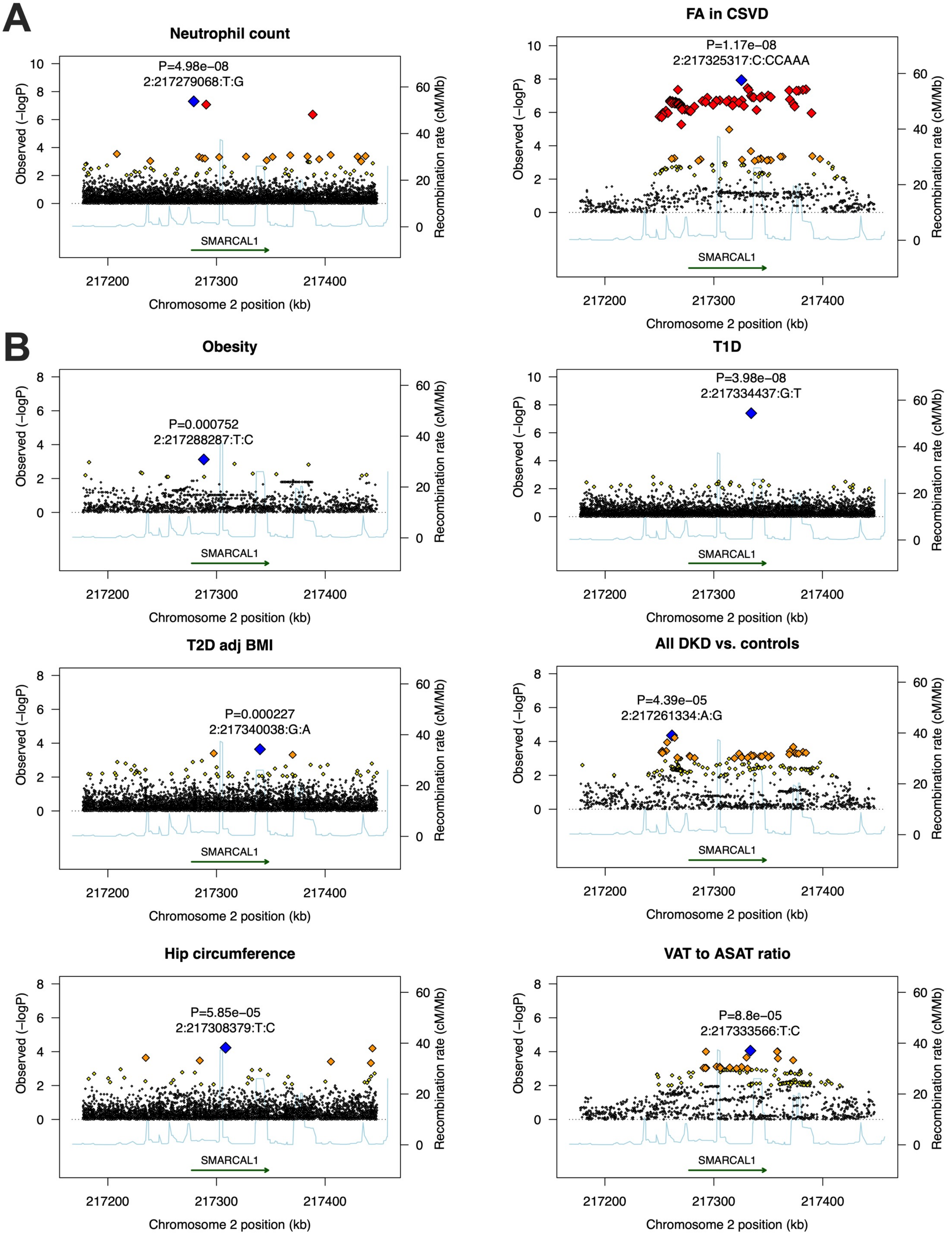


**Fig. S6. Genetic association analysis of the variants at the SMARCAL1 gene locus with human phenotypes.** Regional association plots of the variants with neutrophil count and fractional anisotropy in cerebral small vessel disease (FA in CSVD) (**A**) and other metabolic syndromes as indicated (**B**) (see Supplementary Data 4 and 5 for raw data, respectively). The p values and variant IDs of the top variants (blue square) for each phenotype are indicated.

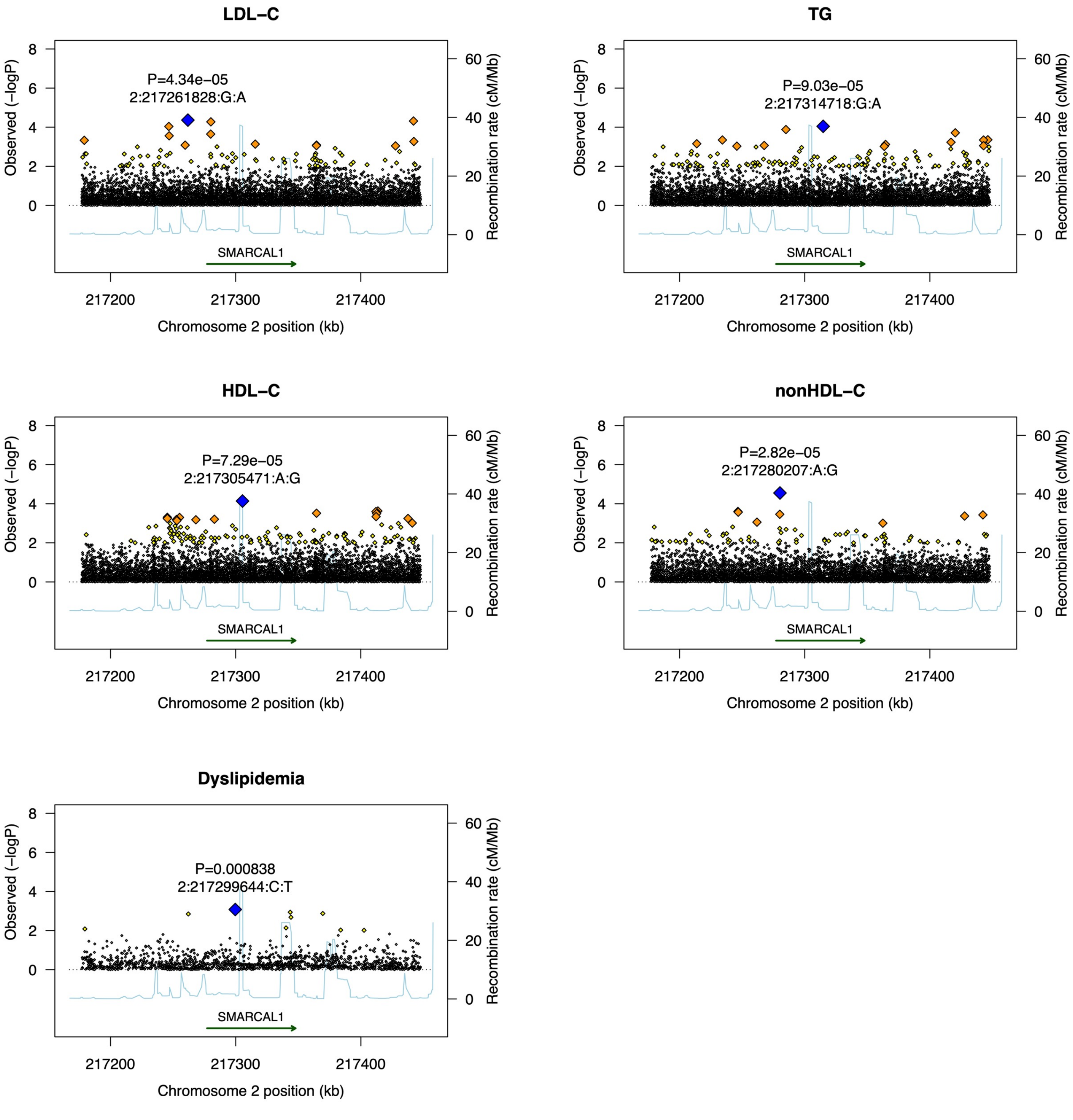


**Fig. S7. Genetic association analysis of the variants at the SMARCAL1 gene locus with lipid phenotypes.** Regional association plots of the variants with lipid phenotypes as indicated (see Supplementary Data 6 for raw data). The p values and variant IDs of the top variants (blue square) for each phenotype are indicated.

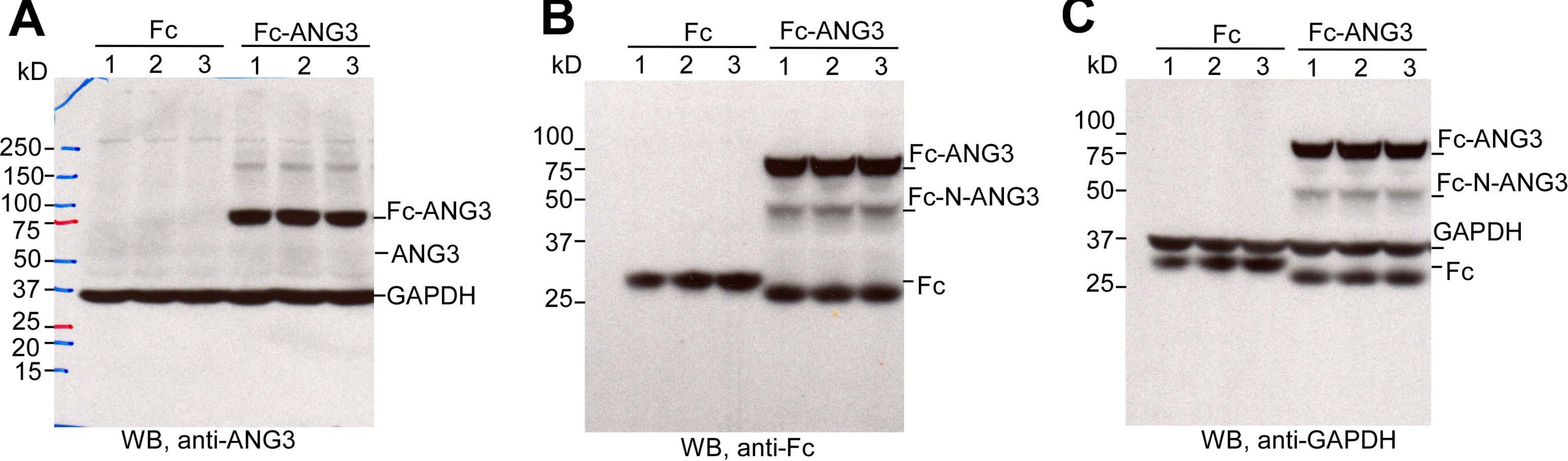


**Fig. S8**. Full images of Figure S1 top (**A**) and bottom (**B** and **C**) panels.

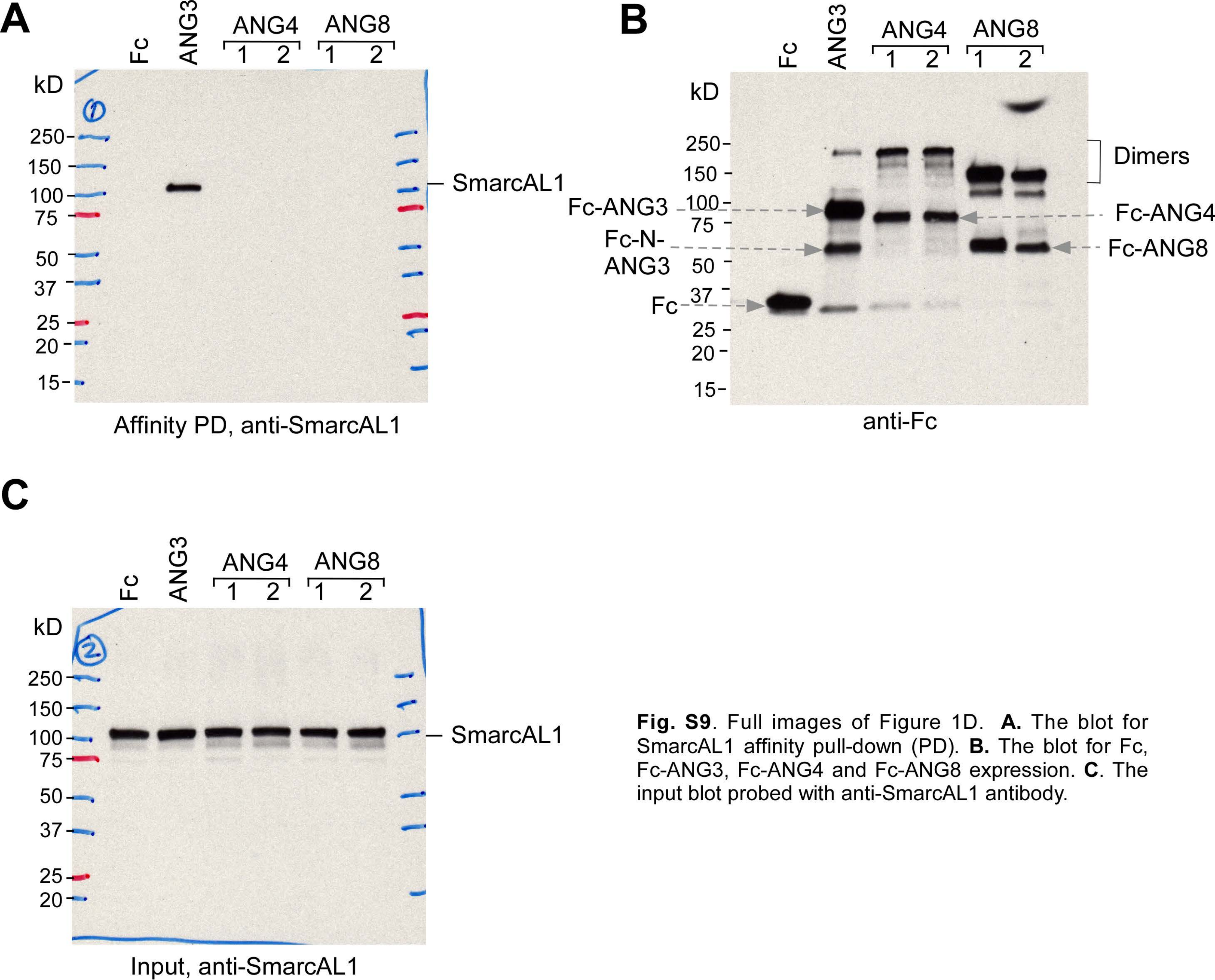


**Fig. S9**. Full images of Figure 1D. **A.** The blot for SmarcAL1 affinity pull-down (PD). **B.** The blot for Fc, Fc-ANG3, Fc-ANG4 and Fc-ANG8 expression. **C**. The input blot probed with anti-SmarcAL1 antibody.

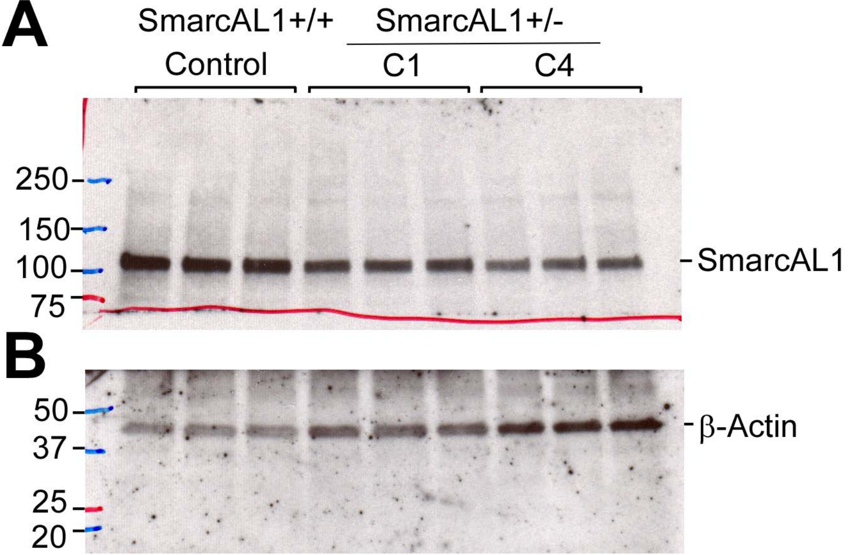


**Fig. S10**. Full images of Figure 2A bottom panel. **A.** SmarcAL1 blot. **B.** β-Actin blot. The whole blot was cut into two parts. The top half was probed with anti-SmarcAL1 antibody (**A**). The bottom half was probed with anti-b-Actin antibody (**B**).

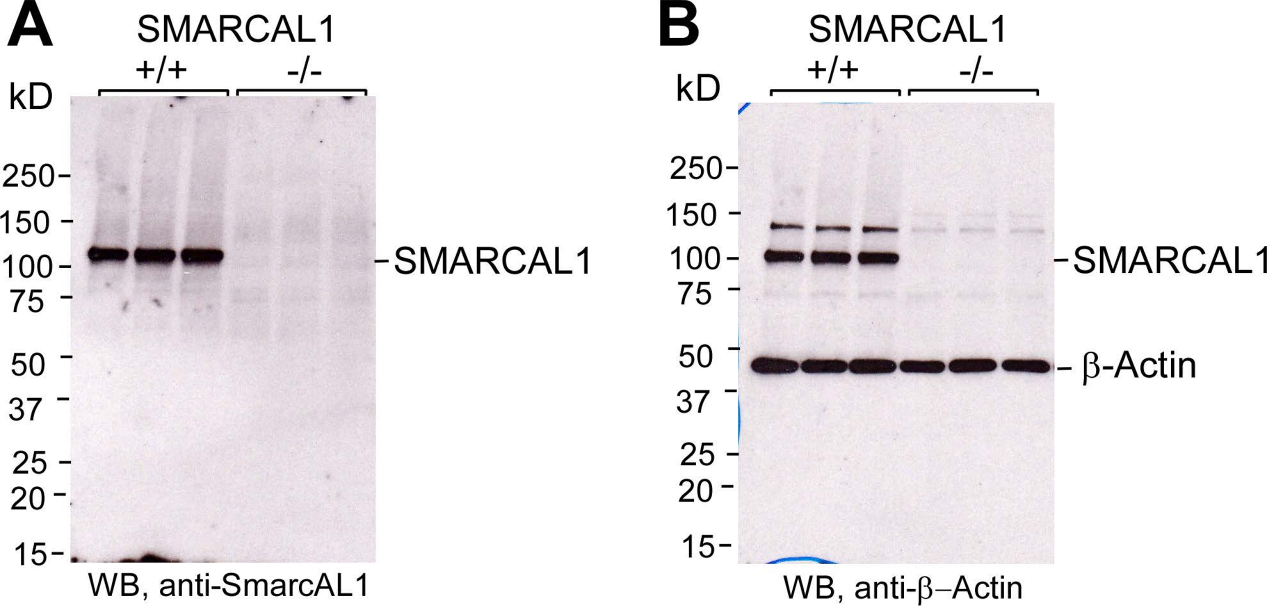


**Fig. S11**. Full images of Figure 2B bottom panel. **A.** SMARCAL1 blot. **B.** β-Actin blot.

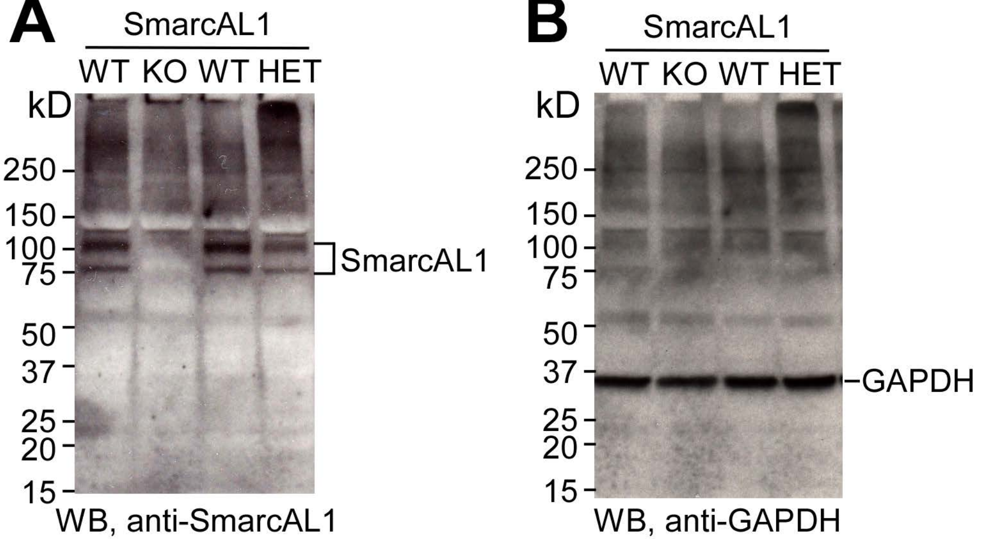


**Fig. S12**. Full images of Figure 2C. **A.** SMARCAL1 blot. **B.** GAPDH blot.

**Supplementary Table 1. Materials used in this study**

| Primary antibodies | Cat # | Suppliers/Manufacturers |
|---|---|---|
| Goat anti-Human IgG (Fc specific) antibody | I2136 | Sigma-Aldrich |
| Goat anti-Human IgG (Fc specific)-FITC antibody | F9512 | Sigma-Aldrich |
| Goat anti-Human IgG (Fc specific)-peroxidase antibody | A0170 | Sigma-Aldrich |
| Mouse monoclonal SmarcAL1 antibody | sc-376377 | Santa Cruz Biotechnology |
| Rabbit polyclonal anti-SmarcAL1 antibody | NBP2-20414 | Novus Biologicals |
| Rabbit polyclonal anti-SmarcAL1 antibody | ABE1836 | EMD Millipore |
| Rabbit polyclonal SmarcAL1 antibody (H-124) | sc-292681 | Santa Cruz Biotechnology |
| Goat polyclonal SmarcAL1 antibody (G-20) | sc-54518 | Santa Cruz Biotechnology |
| Rabbit polyclonal SmarcAL1 antibody | A301-616A | BETHYL Laboratories |
| Rabbit polyclonal anti-SmarcAL1 antibody | ab115611 | abcam |
| Rabbit polyclonal SmarcAL1 antibody | NBP2-20414 | Novus Biologicals |
| Rabbit monoclonal Angptl3 (EPR7319) antibody | ab126718 | abcam |
| Mouse monoclonal Angptl3 (G-5) antibody | sc-365887 | Santa Cruz Biotechnology |
| Rabbit polyclonal anti-ANGPTL3 antibody | ab154009 | abcam |
| Rabbit polyclonal anti-PMP70 antibody | PA1-650 | Thermo Fisher Scientific |
| Mouse monoclonal anti-PMP70 antibody | SAB4200181 | Sigma |
| Rabbit monoclonal ATP5A [EPR13030(B)] antibody | ab176569 | abcam |
| Mouse monoclonal RPA1 (4C4) antibody | MA517166 | Thermo Fisher Scientific |
| Mouse monoclonal β-actin antibody (C4) (HRP conjugated) | sc-47778 | Santa Cruz Biotechnology |
| Rabbit monoclonal GAPDH (14C10) (HRP conjugate) antibody | #3683 | Cell Signaling Technology |
| Goat polyclonal anti-ApoB antibody | AB742 | Millipore |
| Mouse monoclonal ApoB (C1.4) antibody | sc-13538 | Santa Cruz Biotechnology |
| **Secondary antibodies** | **Cat #** | **Suppliers/Manufacturers** |
| Goat anti-mouse IgG (whole molecule) - peroxidase antibody | A4416 | Sigma-Aldrich |
| Goat anti-mouse IgM (m-chain specific) - peroxidase antibody | A8786 | Sigma-Aldrich |
| Goat anti-rat IgG (whole molecule) - peroxidase antibody | A9037 | Sigma-Aldrich |
| Goat anti-rabbit IgG (H+L) cross-adsorbed secondary antibody, alexa fluor 488 | A11008 | Fisher Scientific |
| Goat anti-mouse IgG (H+L) cross-adsorbed secondary antibody, alexa fluor 546 | A11003 | Fisher Scientific |
| Donkey anti-rabbit IgG (H+L) highly cross-adsorbed secondary antibody, alexa fluor 488 | A21206 | Fisher Scientific |
| Donkey anti-rabbit IgG (H+L) highly cross-adsorbed secondary antibody, alexa fluor 555 | A31572 | Fisher Scientific |
| Donkey anti-mouse IgG (H+L) highly cross-adsorbed secondary antibody, alexa fluor 488 | A21202 | Fisher Scientific |
| Novex goat anti-rabbit IgG (H+L) secondary antibody, alexa fluor 488 conjugate | A11008 | Life Technologies |
| Goat anti-mouse IgG (H+L) highly cross-adsorbed secondary antibody, alexa fluor 568 | A11031 | Fisher Scientific |
| Donkey anti-goat IgG (H+L) cross-adsorbed secondary antibody, alexa fluor 633 | A21082 | Fisher Scientific |
| Goat anti-mouse IgG H+L secondary antibody, alexa fluor 488 | ab150113 | abcam |
| Goat anti-mouse IgG (H+L) secondary antibody, alexa fluor 568 | A11031 | Life Technologies Corporation |
| Donkey anti-goat IgG (H+L) secondary antibody, alexa fluor 633 | A21082 | Life Technologies Corporation |
| **Lipid reagents and assay kits** | **Cat #** | **Suppliers/Manufacturers** |
| Glycerol standard | G7793 | Sigma-Aldrich |
| Cholesterol standard | 1012-030 | Fisher Scientific |
| Human LDL | BT-903 | Thermo Fisher Scientific Chemicals |
| Human Dil-VLDL | J65568-AMH | VWR International |
| Human Dil-LDL | J65330-AMH | Fisher Scientific |
| Human plasma Dil-LDL | L3482 | Life technologies |
| Human plasma Dil-VLDL (BT-922) | J65568 | Alfa Aesar |
| Human plasma Dil-LDL (BT-904) | J65330 | Alfa Aesar |
| BODIPY 558/568 c12 lipid probes | D-3835 | Thermo Fisher Scientific |
| Triglyceride colorimetric assay kit | 10010303 | Cayman Chemical Company |
| Cholesterol fluorometric assay Kit | 10007640 | Cayman Chemical Company |
| Free fatty acid fluorometric assay Kit | 700310 | Cayman Chemical Company |
| Free fatty acid quantification assay kit (colorimetric/fluorometric) | ab65341 | abcam |
| Human ApoB ELISAPRO kit | 3715-1HP-2 | MABTECH |
| **Human primary hepatocytes** | **Cat #** | **Suppliers/Manufacturers** |
| Human primary hepatocytes | HMCPMS | Life Technologies/Thermo Fisher |
| **Cell culture media and serum** | **Cat #** | **Suppliers/Manufacturers** |
| Dulbecco's Modified Eagle Medium (DMEM) | 11995 | Thermo Fisher Scientific |
| Opti-MEM™ I Reduced Serum Medium | 31985070 | Thermo Fisher Scientific |
| FBS (heat inactivated) | 12306C | SAFC |
| Low IgG FBS | 100120 | GEMINI Bio-product |
| Super low igG FBS | SH30898.03 | VWR International |
| Absolute low IgG FBS | 100-120 | Fisher Scientific |

| Mouse diets | Cat # | Suppliers/Manufacturers |
|---|---|---|
| Mouse high fat diet | TD.06414 | ENVIGO |
| Rodent diet with 45 kcal% fat | D12451 | Fisher Scientific |

| Western blotting assay reagents and buffers | Cat # | Suppliers/Manufacturers |
|---|---|---|
| Protran membrane | 10439194 | Westnet |
| Amersham Enhanced chemiluminescence (ECL) detection | RPN2209 | Genesee Scientific |
| Membrane nitrocellulose (0.45 $\mu$M) | 1620115 | Bio-Rad Laboratories |
| Nonfat dry milk | M-0841 | Fisher Scientific |
| Tris-glycine SDS running buffer (10X) | BP-150 | Boston Bioproducts |
| Tris Buffered Saline (TBS) (10X) | BM-301 | Boston Bioproducts |
| Transfer buffer 10X | BP-190 | Boston Bioproducts |
| Mini-PROTEAN TGX SDS-PAGE | 4561095 | Bio-Rad Laboratories |

| Immunofluorescence reagents | Cat # | Suppliers/Manufacturers |
|---|---|---|
| Mounting media | P36931 | Vector Laboratories |
| GG-18-collagen coverslips | GG-18-Collagen | neuvitro.com |
| Polylysine cover glass | GG18PLL | Fisher Scientific |

| Chemical and biological reagents and buffers | Cat # | Suppliers/Manufacturers |
|---|---|---|
| DMSO | D8418 | Sigma-Aldrich |
| Isoflurane | 10019-360-40 | Baxter Healthcare |
| IGPAL-CA-630 | I8896 | Sigma-Aldrich |
| Methanol optima LC/MS grade | A456-4 | Fisher Scientific |
| Isopropanol, HPLC grade | BP26324 | Fisher Scientific |
| Heparin sodium salt | H3393 | Sigma |
| Doxycycline hyclate | D9891 | Sigma-Aldrich |
| Geneticin selective antibiotic (G418) | 10131-035 | Life Technologies |
| Purimycin dihydrochloride | A11138-03 | Life Technologies |
| Zeocin | ant-zn-05 | InvivoGen |
| Micrococcal nuclease | 88216 | Thermo Scientific |
| Novex mouse IgG1 isotype control | 26100 | Life Technologies |
| Normal Goat Serum | 31872 | Thermo Fisher Scientific |
| Mouse IgG1 Isotype control | 02-6100 | Thermo Fisher Scientific |
| Pierce normal human serum | 31876 | Thermo Fisher Scientific |
| TAE (50X) | BM-250 | Westnet |
| PBS (10X) | BM-220 | Boston Bioproducts |
| Paraformaldehyde solution 4% in PBS | sc-281692 | Santa Cruz Biotechnology |
| Glycine buffer 0.2M (pH 3.0) | BB-95-2 | Fisher Scientific |

| Molecular biology reagents and kits | Cat # | Suppliers/Manufacturers |
|---|---|---|
| Endozero plasmid maxi prep | D4205 | Fisher Scientific |
| Lipofectamine 2000 Reagent | 11668-027 | Invitrogen |
| RNeasy plus mini kit | 74134 | Qiagen |
| Platinum Taq DNA polymerase, high fidelity | 11304-011 | Life Technologies |
| T7 endonuclease | M0302L | New England BioLabs |

| CRISPR gene editing reagents and plasmids | Cat # | Suppliers/Manufacturers |
|---|---|---|
| pX602 | 61593 | Addgene |
| pCAG-EGXXFP | 50716 | Addgene |
| pSpCas9(BB)-2A-Puro (PX459) V2.0 | #62988 | Addgene |
| Sureyor mutation kit S100 | 706020 | Fisher Scientific |
| Cap9 protein | CP01 | PNA Bio |
| MEGAclear Kit for purification for large scale transcritpion reactions | AM1908 | Ambion/Life Technologies |
| pCas-Scramble | GE100003 | OriGene |

| Protein purification and assay reagents | Cat # | Suppliers/Manufacturers |
|---|---|---|
| Immobilized rProtein A | IPA-400HC | RepliGen |
| Imperial protein stain | 26615 | Thermo Fisher Scientific |
| Pierce™ BCA Protein Assay Kit | 23227 | Thermo Fisher Scientific |
| Complete EDTA-free protease inhibitor | 11873580001 | Roche Diagnostics |
