# Supplementary material for: Chromatin regulator SMARCAL1 modulates cellular lipid metabolism
Source: Commun Biol. 2023 Dec 21;6:1298. doi: 10.1038/s42003-023-05665-6 (PMC10739977; doi:10.1038/s42003-023-05665-6)
Supplement: Supplementary file 2 — Description of Additional Supplementary Files [file 42003_2023_5665_MOESM2_ESM.pdf]

Description of Supplementary Data Files

File name: Supplementary Data 1
Description: Metabolomics analysis of cell lipid and polar lipid metabolite changes of heterologous *SmarcAL*1$^{+/-}$ McA cells as compared with WT control cells (also see Supplementary Figure S2).

File name: Supplementary Data 2
Description: Metabolomics analysis of cell lipid and polar lipid metabolite changes of homologous *SmarcAL1*$^{-/-}$ Huh7 cells as compared with WT control cells (also see Supplementary Figure S3).

File name: Supplementary Data 3
Description: Inactivation of *SmarcAL1* gene decreases the expression of genes related to cellular lipid metabolism. Differentially expressed genes related to lipid, FA, mitochondria, peroxisome metabolism and insulin resistance from RNA-seq analysis (also see Figure 4C).

File name: Supplementary Data 4
Description: Genetic association analysis of the genetic variations at the SMARCAL1 gene locus with neutrophil count and FA in CSVD (also see Supplementary Figure S6A).

File name: Supplementary Data 5
Description: Genetic association analysis of the genetic variations at the SMARCAL1 gene locus with metabolic syndromes (also see Supplementary Figure S6B).

File name: Supplementary Data 6
Description: Genetic association analysis of the genetic variations at the SMARCAL1 gene locus with lipid phenotypes (also see Supplementary Figure S7).
